# Biosensing technologies for foodborne pathogen detection and healthcare: principles, emerging materials, and intelligent platforms

**DOI:** 10.1007/s00604-026-07976-x

**Published:** 2026-03-10

**Authors:** Purshottam J. Assudani, Balakrishnan P, Anny Leema A, Gina George, Ankita Avthankar, Aditya Tiwari, Manish Bhaiyya, Madhusudan B. Kulkarni

**Affiliations:** 1 School of Computer Science and Engineering, Ramdeobaba University, Nagpur, 440013 India; 2 School of Computer Science and Engineering, Vellore Institute of Technology, Vellore, 632014 India; 3 Department of Computer Applications, Auxilium College, Vellore, 632014 India; 4 Symbiosis Institute of Technology, Nagpur Campus, Symbiosis International (Deemed University), Pune, 411057 Maharashtra India; 5 School of Engineering and Applied Science, Ahmedabad University, Navrangpura, Ahmedabad, Gujarat 380009 India; 6 Department of Chemical Engineering and the Russell Berrie Nanotechnology Institute, Technion Israel Institute of Technology, Haifa, 3200003 Israel; 7 Department of Electronics and Telecommunication, Shri Sant Gajanan Maharaj College of Engineering, Shegaon, 444203 India; 8 Manipal Institute of Technology, Manipal Academy of Higher Education (MAHE), Manipal, 576104 India

**Keywords:** Foodborne pathogens, *Escherichia coli*, *Salmonella*, *Listeria monocytogenes*, Biosensors, Electrochemical sensing, Optical sensing, Artificial intelligence, Machine learning, Nanomaterials, Food safety monitoring, Point-of-care-testing, Health diagnosis

## Abstract

Foodborne pathogens such as *Escherichia coli* (E. Coli), *Salmonella*, and *Listeria* monocytogenes continue to pose a major potential threat to global public health and therefore rapid, accurate, and field-deployable detection methods are still extremely desirable. This review describes cutting-edge examples of advanced biosensing platforms for the strategy of detecting these priority pathogens, focusing on clinical detection and highlighting electrochemical, optical, and microfluidic sensing modalities. This has been enabled by recent advances in functional nanomaterials, molecular recognition elements (including aptamers and nanozymes), and surface engineering strategies rendering sensors much ‘smarter’/improved in terms of sensitivity, specificity, and behaviour towards complex food matrices. However blending these biosensors with artificial intelligence (AI) and Machine Learning (ML) enabled intelligent pattern recognition, real-time analytics, and multiplexing at high-speed, turning traditional detection systems into smart diagnostic devices. We critically review recent case studies in light of biosensor design, signal transduction mechanisms, models of AI, performance validation, and applicability in different food environments. The principal challenges are identified which include matrix interference, instability of biorecognition elements, limitations in scalability, and the need for regulatory standardization. We discuss these with associated mitigation strategies that are technically sound, including ratiometric sensing, microfluidic pre-treatment techniques, explainable AI, and printable electronics. Forward-looking, we discuss biosensors enabled by being self-powered, biosensor hubs with modular pathogen panels, blockchain incorporation, and standardized validation pipelines. This review offers a prospective view toward enabling intelligent, robust, and regulation-ready biosensing platforms for next-generation food safety monitoring through the bridging of technological innovations with practical implementation.

**Graphical Abstract:**

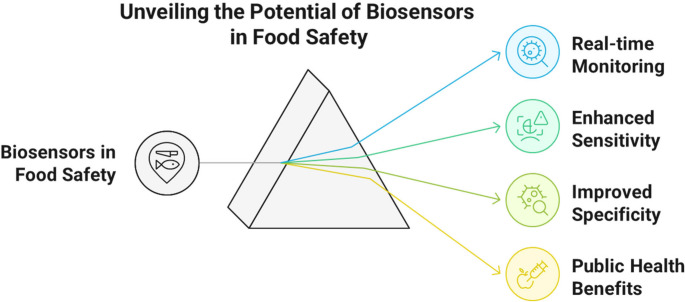

## Highlights

Foodborne illnesses caused by E. coli, Salmonella, and Listeria monocytogenes remain a global public health concern, driving the demand for rapid, accurate, and field-deployable detection strategies.

This review comprehensively explores advanced biosensing platforms tailored for detecting these priority pathogens, highlighting progress in electrochemical, optical, and microfluidic sensing mechanisms.

Integrating functional nanomaterials, molecular recognition elements such as aptamers and nanozymes, and surface engineering techniques has significantly enhanced sensor sensitivity, specificity, and adaptability to complex food matrices.

Moreover, the convergence of biosensors with AI and ML has enabled intelligent pattern recognition, real-time analytics, and high-throughput multiplexing.

Key challenges, including matrix interference, bioreceptor instability, manufacturing scalability, and regulatory standardization, are discussed.

## Introduction

Foodborne illness caused by bacterial pathogens is a worldwide, ongoing, and evolving public health problem. The millennial estimates place more than 600 million cases of illnesses and thousands of deaths resulting from contaminated food; among the bacteria, *E. coli*,* Salmonella*, and *Listeria monocytogenes* are some of the most common implicated agents [1, 2]. These pathogens are responsible for causing acute gastroenteritis, long-term complications, and thousands of deaths each year, most of which affect the most susceptible, such as infants, pregnant women, the elderly, and those who are immunocompromised [3]. This makes early detection and intervention difficult to achieve due to the ubiquity of these pathogens in a multitude of food matrices, such as leafy greens, dairy products, seafood, and processed meats. As food supply chains continue to become more globalised and decentralised, the need for early, reliable and on-site detection of hazards is paramount. Both challenges highlight the need for robust detection strategies that can span all food matrices and stages of the supply-chain [4, 5].

Traditional detection methods include standard pathogen detection methods, especially culture-based bacterial detection, ELISA, and PCR. However, traditional methods have also many weaknesses to be applied in practice. For example, culture methods are very specific but lengthy and laborious, requiring 2–5 days to obtain. Molecular methods like PCR and qPCR while faster still require skilled operators, expensive instrumentation, and exacting sample preparation protocols thereby excluding them from resource-poor laboratory or field-deployable applications [6–8]. Further, these methods might be subject to interference from matrix components; yield false negatives at low bacteria counts; and, in most cases, have limited utility in allowing multiplexing or real-time monitoring. These limitations highlight the continuing demand for newer, faster, more sensitive, cheaper means of detection that can be applied in the farm-to-fork continuum [9]. Consequently, alternative analytical approaches that combine laboratory-level accuracy with operational simplicity and speed are increasingly sought.

Accordingly, biosensing technologies have emerged as prime candidates to fill the gap between lab-like accuracy in a ready-for-field application [10–13]. Typical biosensors comprise a biological recognition element - antibody, aptamer, bacteriophage, CRISPR-Cas system, etc. - and a transducer - electrochemical, optical, piezoelectric, etc. - which converts the biorecognition event to a measurable signal [14–17]. These detection platforms are characterized by high specificity, portability, relatively fast response times (< 30 min most of the time), and opportunities for miniaturization and automation. Their compatibility with microfluidic, paper-based, and smartphone-based detection systems adds to their acceptability regarding decentralized diagnostics of food safety [18, 19]. While these features address many practical limitations of traditional methods, continued performance improvements depend strongly on advances in materials and system integration.

More recently, the performance and versatility of bacterial biosensors has been improved by advances in materials science and nanotechnology. Many types of nanomaterials (gold nanoparticles (AuNPs), carbon nanotubes (CNTs), graphene oxide (GO), quantum dots (QDs),, MXenes, etc.) have been investigated with regard to their signal amplification properties, biocompatibility and large surface area to volume ratio for possible use in the biosensor platform [20]. These materials have improved the detection limits to often below 10 CFU/mL sensitivity, screen several pathogens by way of multiplexing, and allow for various modes of device fabrication [21]. Functional materials such as nanozymes, responsive hydrogels, and molecularly imprinted polymers show enhanced stability and selectivity and enable storage over extended periods of time under varying environmental conditions [22]. Moving further than the biosensing surface itself, biosensing technologies increasingly are integrated into designed smart systems and digital technologies. Microfluidic lab-on-a-chip platforms operate in automated sample enrichment, mixing, and detection in compact, user-friendly formats [23, 24]. Meanwhile, smartphone readers, Bluetooth-enabled transducers, and cloud-based information dashboards turn biosensors into IoT devices that enable remote monitoring, real-time alerts, and data aggregation in a centralized manner. AI and ML algorithms also ensure enhanced signal interpretation, noise filtering, pattern recognition, and predictive analytics, due to which smart food safety systems can not only identify microbial contamination but predict it, too [25, 26]. Despite these technological advancements, existing literature often treats these components independently, limiting a holistic understanding of deployable biosensing systems.

While many recent review articles have addressed the topic of biosensing for foodborne pathogens, most have been narrow in their focus on a single pathogen, a single platform, or with scant insight into systems-level integration. Typically, as in those papers in [27, 28], new achievements of electrochemical biosensors regarding Listeria monocytogenes detection or outline certain outbreakdriven needs of rapid detection technologies; these studies generally focus narrowly on one or a few pathogens or selected detection techniques.

Similarly, other works such as [29–31] provide focused accounts of Salmonella or E. coli control strategies, including the use of probiotics, aptasensors, or SERS-based detection, but lack integration across pathogen types or technological platforms. Additional reviews like [7, 32, 33] emphasize multiplexed systems or hybrid biosensors, yet stop short of a systems-level perspective. Our review, on the other hand, represents a pathogen-transcending, platform-agnostic synthesis that systematically organizes this fast-evolving field of bacterial biosensing based on a unified framework centered on biosensor principles, material innovations, such as nanomaterials, nanozymes, and CRISPR systems, and real-world deployment strategies, including smartphone integration, AI/ML tools, and IoT connectivity. It uniquely integrates concepts of the future, such as blockchain-based traceability, wearable sensors, and federated AI for decentralized monitoring. Within this framework, strategies for the identification of E. coli, Salmonella, and Listeria are contextualized, framed within a comparative cross-cutting biosensing architecture that fills the gap between analyses that are pathogen-specific or siloed in stand-alone technologies and presents a holistic, futuristic roadmap toward transitioning food safety from reactive inspection to predictive intelligence. Finally, to explicitly clarify the novelty of the proposed integrative framework, Table 1 compares this review with representative recent reviews in terms of pathogen coverage, sensing modalities, materials, system-level integration, and deployment considerations.

**Table 1:** Comparison of representative recent reviews vs. this review

Review (Year)	Main Scope	What it covers well	What it lacks (vs. this integrative framework)
Silva et al. (2020), [27]	Listeria-focused electrochemical biosensing	Electrochemical strategies for Listeria in foods	Not multi-pathogen; limited system-level integration (AI/IoT, deployment)
Cossettini et al. (2022), [28]	Multi-pathogen biosensor detection	Broad biosensor coverage for major pathogens	Limited emphasis on AI/ML, smartphone/IoT, and framework-style synthesis
Kabiraz et al. (2023), [7]	Conventional and advanced detection methods	Wide overview of culture, PCR, and newer methods	Descriptive; not structured as materials → platform → deployment framework
Aladhadh (2023), [32]	Modern methods for foodborne pathogens	Broad coverage of detection approaches	Limited focus on emerging materials and AI-enabled biosensing
Petrucci et al. (2021), [4]	On-site detection of food and waterborne bacteria	Strong field and on-site detection perspective	Not centered on E. coli/Salmonella/Listeria triad or AI-driven convergence
Kim & Kim (2021), [34]	Conventional and alternative detection methods	Clear comparison of classical vs. alternative techniques	Not biosensor-material driven; limited future-ready architecture
Kulkarni et al. (2023),[23]	Microfluidics-based electrochemical sensors	Integration of microfluidics and electrochemical sensing	Not pathogen-triad focused; lacks full-stack integration
Puiu & Bala (2020), [24]	Microfluidics-integrated biosensing platforms	Comprehensive lab-on-chip overview	No unified integration of materials, AI/ML, smartphone/IoT, and validation
Abdelbasset et al. (2023), [25]	Smartphone-based aptasensors	Smartphone-enabled sensing strategies	Device-centric; limited regulatory and multi-pathogen framework
Qin et al. (2025), [26]	Smartphone-based biosensors for food testing	State-of-the-art smartphone readout strategies	Does not unify biosensing principles, materials, AI/ML, and deployment readiness
This Review	E. coli, Salmonella, Listeria across biosensors, materials, AI/ML, smartphone/IoT	Pathogen-transcending, platform-agnostic synthesis from design to deployment	—

It is important to assess published sensing strategies according to agreed-upon analytical parameters rather than just employing descriptive comparisons in order for the rapidly expanding biosensing literature to be interpreted in a uniform way across different platforms. Accordingly, throughout this review, biosensing platforms are interpreted using a unified analytical framework that considers: (i) sensitivity and limit of detection (LOD) aligned with regulatory thresholds, (ii) specificity and resistance to cross-reactivity within complex microbial backgrounds, (iii) response time and enrichment dependence, (iv) tolerance to food-matrix interference and robustness in real samples, (v) quantification reliability and susceptibility to analytical drift or false outcomes, (vi) reproducibility and validation readiness across batches and operational settings, and (vii) practical deployability, including portability, workflow simplicity, and cost considerations. The establishment of these criteria at the outset enables meaningful comparisons of electrochemical, optical, microfluidic, smartphone-enabled, and A.I.-enabled biosensing devices, thus supporting the integrated and cross-cutting approach developed in subsequent sections.

Finally, in keeping with this theme, this review is structured to move progressively from biological context to real-world deployment considerations to assist clarity, fluency, and navigation by the reader’s brain. In Sect.  2 we touch on the priority foodborne pathogens (Escherichia coli, Salmonella spp., and Listeria monocytogenes) and the routes of their contamination the farm-to-fork continuum and setting the biological and regulatory context for their detection. Section  3 provides an overview of the core biosensing principles of biorecognition elements and transduction mechanisms and key performance metrics. Section  4 covers material-level innovations, including emerging nanomaterials, smart polymers, bioengineered receptors, and enzyme mimics that enhance sensitivity, selectivity, and stability. Section  5 covers system-level integration comprising microfluidic and lab-on-a-chip platforms, smartphone- and IoT-enabled sensing architectures, and AI/ML-assisted biosensing frameworks that enable intelligent, decentralized diagnostics. Finally, Sect.  6 consolidates the deployment-relevant challenges inclusive of robustness in complex food matrices, reproducibility and stability of biological components, scalability, cost, and regulatory considerations-along with corresponding mitigation strategies. The structured organization will, thus, enable readers to coherently move from pathogen biology and biosensor design fundamentals to practical implementation pathways for next-generation food safety and healthcare-relevant biosensing platforms.

## Overview of Foodborne Pathogens and Contamination Routes

Foodborne illness remains a significant global public health challenge, leading to an estimated 600 million cases of illness and approximately 420,000 deaths every year around the world, putting strain on health services and food supply chains (https://www.who.int/activities/estimating-the-burden-of-foodborne-diseases). Amongst bacterial hazards, *E. coli*, *Salmonella spp*. and *Listeria* are almost always priority pathogens as results of their common occurrence, association with outbreaks and capacity to cause severe or even fatal disease. *Salmonella* causes millions of infections each year in both rich countries and poorer countries, while Shiga toxin–producing *E. coli* (STEC, a form of E. coli) causes more than 10% of foodborne outbreaks associated with meat, fresh produce, and water. In comparison to these, *Listeria* monocytogenes is rarer but has extremely high hospitalization and death rates, especially often sadly, among pregnant women, new babies, the elderly, and immunocompromised people, though despite the best efforts of food standards agencies to control it, most of the risks don’t seem to be going away [35–37].

Understanding the biologically distinct epidemiology, transmission, and risk associated with these pathogens is fundamental to a pathogen-specific approach to detection and monitoring strategies. Accordingly, we provide in the following subsections, overviews of (i) *E. coli*, emphasizing infectious dose and outbreak epidemiology, (ii) Salmonella spp., highlighting persistence in foods and food processes, and (iii) Listeria monocytogenes, detailing its persistence in refrigeration and biofilms. This overview provides the biological and contamination context for discussing various biosensing methodologies, described subsequently.

### Escherichia Coli (E. coli)

Pathogenic strains of *E. coli*, particularly E. coli O157:H7, are responsible for severe gastrointestinal infections and life-threatening complications such as hemolytic uremic syndrome [38, 39]. These Shiga toxin-producing E. coli strains are commonly transmitted via undercooked ground beef, unpasteurized dairy, raw produce, and contaminated water. Infection may still occur at very low doses (10–100 CFU) of O157:H7, causing illness prompting the need for detection [40]. Globally, outbreaks have been associated with leafy greens, sprouts, and fresh juices, often linked to pre-harvest contamination from livestock or irrigation water [41, 42].

### Salmonella spp

*Salmonella* remains one of the major causes of bacterial foodborne illnesses worldwide, causing millions of infections and thousands of deaths every year [43]. Non-typhoidal *Salmonella* enterica serovars, including Enteritidis and Typhimurium, are most often implicated. These pathogens have widely been isolated in different kinds of foods, ranging from raw poultry and eggs to dairy, meat, nuts, and processed foods [35, 44]. Infections generally lead to gastroenteritis, though it may result in invasive diseases among immunocompromised patients. Unlike E. coli, Salmonella can survive under dry conditions, allowing for its persistence in both powdered foods and its survival in food-processing environments. Large-scale outbreaks have drawn attention to the need for sensitive, rapid, and on-site detection in food chains [45, 46].

### Listeria Monocytogenes

*Listeria* monocytogenes causes listeriosis, an uncommon but severe infection with high mortality among pregnant women, neonates, the elderly, and immunocompromised individuals. This pathogen represents a particular concern in processed ready-to-eat foods, soft cheeses, smoked seafood, and deli meats because it differs from most foodborne pathogens in that it can grow at refrigeration temperatures. Resilient biofilm development and sanitizer resistance further complicate control of Listeria within food processing environments. In addition to morbidity and mortality, outbreaks caused by Listeria often involve large recalls and economic losses, emphasizing the need for real-time monitoring and stringent detection standards [47, 48].

### Contamination Routes from Farm to Fork

Foodborne pathogens may be introduced to food supplies at virtually every point along the way in the growing and processing of (i.e. “farm-to-fork”) food products. Foodborne pathogens may be introduced on-farm, during processing, during shipping/distributing, at retail store and consumer/household preparation. These routes are important to grasp in a structured way to inform effective biosensing strategies for early detection, risk mitigation and real time monitoring.

#### Pre-harvest Contamination (On-farm Stage)

Contamination at the farm generally occurs via environmental reservoirs - soils, waters, livestock, and wildlife. Pathogenic contamination, particularly *E. coli* and *Salmonella*, can be spread through the application of contaminated manure and inadequately treated biosolids used as fertilizers. Irrigation of fresh produce, notably for leafy greens and sprouts with untreated surface waters further spreads pathogens onto the fresh produce. Direct contact between crops and livestock also contributes significantly to pre-harvest contamination. Importantly, these contamination events often occur at low bacterial loads, making early-stage detection challenging but critically important [49–51].

#### Harvesting and Primary Handling

During harvesting, pathogens may be transferred via contaminated harvesting and storage equipment as well as worker hands or gloves; mechanical damage to the produce surfaces during harvesting further promotes bacterial attachment and internalization. In the production of meat and poultry, contamination may occur during the slaughtering and evisceration processes when material from the intestines come into contact with carcasses. At this stage, cross-contamination events can rapidly amplify pathogen spread across large food batches [52].

#### Processing and Manufacturing

Perhaps the most serious amplification point for contamination is in food processing environments that utilize common equipment, conveyor belts, cutting tools and contact surfaces. *Listeria* monocytogenes and other pathogens are problematic in processing and food preparation facilities because of their propensity to form biofilms on stainless steel, plastic and rubber surfaces and to persist despite routine cleaning. Inadequate heat treatment, improper pasteurization, or post-process contamination can lead to the introduction of pathogens to otherwise safe food products. Ready-to-eat (RTE) foods are especially vulnerable at this stage because no further kill steps are applied before consumption [53–55].

#### Storage, Transportation, and Cold-chain Distribution

While stored and in transit, lapses in temperature and humidity control as well as packaging and handling can create opportunities for pathogens to survive or grow. Because *Listeria* can grow at refrigeration temperatures, cold-chain distribution of foods such as dairy products, deli meats, and seafood can be associated with listeriosis. Cross-contamination may be a concern during bulk transportation if containers and vehicles are not adequately cleaned between shipments. These stages highlight the need for continuous or periodic monitoring, rather than single-point testing [56, 57].

#### Retail, Food Service, and Consumer Handling

Beyond the factory, at the retail and consumer levels, risks of contamination exist associated with poor storage, insufficient cooking time or temperature, and inadequate hygiene practices. Possible cross-contamination between raw and cooked foods (for example, same cutting board, utensils, surfaces). In the domestic sphere, lack of awareness and knowledge as well as lack of a testing regime further increases exposure. Notably, contamination at this stage directly impacts public health outcomes, underscoring the importance of accessible, rapid, and user-friendly detection tools [58, 59].

#### Implications for Biosensing and Monitoring Strategies

**Fig. 1:**
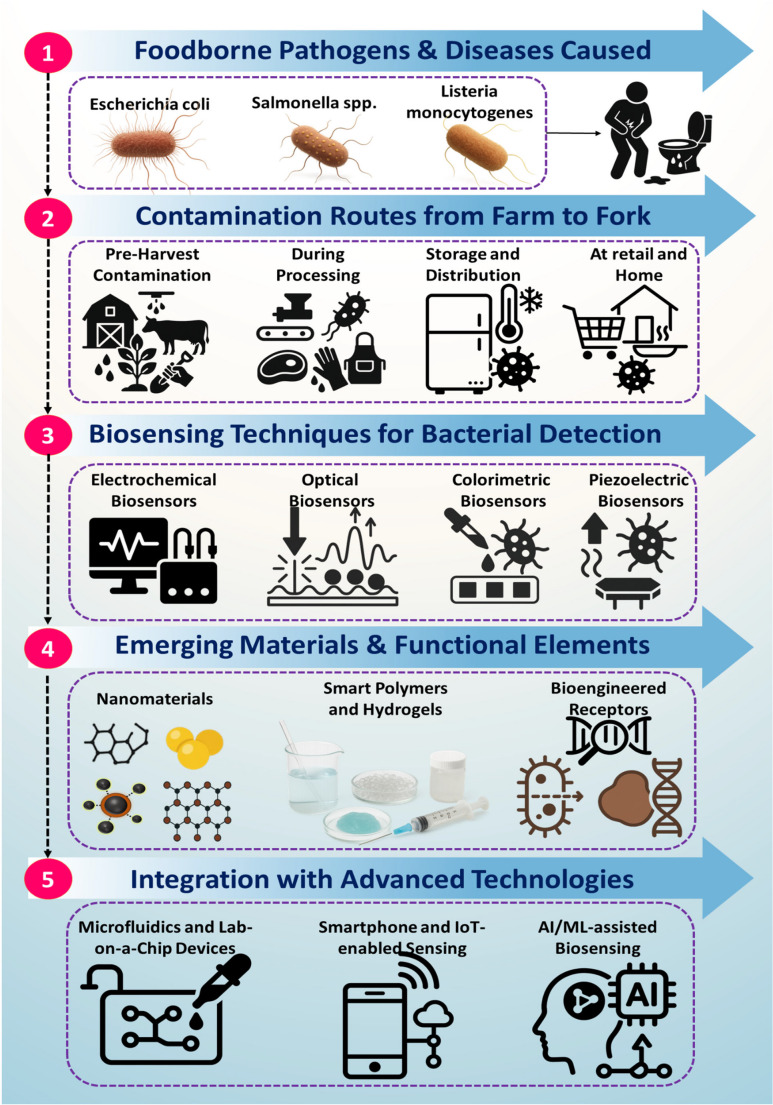
Graphical representation of foodborne bacterial pathogens and their contamination routes from farm to fork, followed by an illustration of the biosensing solutions, detection strategies, and functional materials used, along with the integration of AI, IoT, microfluidics and more

The multi-component of contamination (eco-)systems in the farm-to-fork chain highlights the potential for distributed rapid and matrix tolerant biosensing platforms. These must be capable of operation across diverse environments impacted by contamination. Pre-harvest levels of the chain would require sensors capable of low-level detections in soil/water. Processing and storage stages could benefit from surface-monitoring in addition to biofilm detection. Retail of produce/consumers at their homes could employ portable biosensors/smartphones for onsite indicative screening of contaminants. Consequently, effective food safety surveillance requires biosensing systems that are adaptable to different contamination routes, food matrices, and operational constraints, reinforcing the need for integrated sensing approaches illustrated in Fig. 1 [60–65].

### Regulatory Limits and Detection Requirements

Regulatory authorities globally set guidelines for the levels of risk posed by food pathogens that can be tolerated, and stipulate region-specific criteria that must be met to assure safe foods, thereby indirectly determining their requirements in terms of detection and acceptable methods of analysis. The U.S. FDA has a zero tolerance policy for serious pathogens such as Salmonella spp., Listeria monocytogenes, and E. coli O157:H7 in ready to eat (RTE) foods, with detection of only a few cells of these pathogens in a 25 g sample being unacceptable. However, under the FSMA the focus for food safety is placed primarily on the need to prevent contamination with such pathogens. In contrast, the European Food Safety Authority (EFSA) uses a risk-based and product-category dependent framework to address the risks associated with Listeria monocytogenes. For some ready-to-eat (RTE) foods that cannot support the growth of L. monocytogenes, EFSA allows levels of these bacteria present in these products at the end of shelf-life to be below 100 CFU/g. However, according to EFSA, there is an absolute prohibition against L. monocytogenes in foods intended for sensitive populations. While the Food Safety and Standards Authority of India (FSSAI) mostly conforms to the Codex Alimentarius guidelines by emphasizing negative criteria for high-risk pathogens based on no detection, the FSSAI has also identified other factors in setting up a biosensor platform and working with countries that have diverse and de-centralised food systems, such as the affordability, scalability and ease of testing. Furthermore, due to the many different regulatory environments, the regulatory differences place significant demand on biosensor platforms offering a combination of Flexibility with Quantification; ultra-low limits of detection; affordability and that are Validation Ready for deployment in several regulatory jurisdictions [66–70]. A comparison of regulatory approaches adopted by FDA, EFSA, and FSSAI and their implications for biosensor performance is summarized in Table 2.

**Table 2:** Comparison of regulatory requirements for major foodborne pathogens across regions

Regulatory authority	Regulatory approach	Typical limits for key pathogens	Sample basis	Key implications for biosensor design
FDA (USA)	Zero tolerance; preventive control–driven (FSMA)	Salmonella, E. coli O157:H7, L. monocytogenes: absence in 25 g (RTE foods)	Qualitative (presence/absence)	Ultra-low LOD (< 1–10 CFU/25 g), high specificity, compatibility with confirmatory methods
EFSA (European Union)	Risk-based; product- and shelf-life–dependent	Salmonella: absence in 25 g; L. monocytogenes: absence in 25 g (high-risk) or < 100 CFU/g (low-risk RTE foods)	Quantitative or semi-quantitative	Quantitative biosensors enabling shelf-life–aware risk assessment
FSSAI (India)	Codex-aligned; hygiene- and feasibility-focused	Salmonella, E. coli O157:H7: absence in 25 g; Listeria: absence in RTE foods (evolving standards)	Predominantly qualitative	Affordable, rapid, portable biosensors suitable for decentralized testing

## Biosensing Principles for Bacterial Detection

Biosensors have become important tools in food safety with the rapid, sensitive, and highly specific identification of foodborne bacterial pathogens, including *Escherichia coli*,* Salmonella spp.*, and *Listeria monocytogenes* [71–73]. These analytical platforms function to connect laboratory-based diagnostics and real-time, on-site monitoring applications. A biosensor consists of a biologically active recognition element, and a signal transduction system [74, 75]. The recognition element binds to the pathogen or biomolecule of interest allowing for a specific interaction. This binding is then translated into a signal either electrically, optically, acoustically or colourimetrically. The ability to translate an event that can be considered biological, to one that can be quantified, forms the basis of where biosensors find that they can be useful along the food supply chain [76, 77].

### Recognition Elements

Biosensors can depend on a very wide variety of biorecognition elements for ensuring selectivity. Among the most common recognitions elements are antibodies due to their high binding affinity and specificity against bacterial surface antigens. In the case of detection of pathogenic bacteria, such as E. coli O157:H7, Salmonella Typhimurium, and Listeria monocytogenes, antibodies have been used as recognition elements in both immunosensors and lateral flow devices [78]. Despite the aforementioned advantages, antibodies may suffer from the problems of stability and lot-to-lot variation. Due to that fact, aptamers, which are synthetic single-stranded DNA or RNA molecules selected with SELEX, i.e., Systematic Evolution of Ligands by EXponential enrichment, have become a new, promising alternative. Aptamers demonstrated thermal stability, allowed chemical modification, and were economically feasible. Aptamer-based biosensors have shown excellent prospects in the trace-level analysis of pathogens in milk, meat, and water [79]. The other novel class of recognition element involves the bacteriophages, viruses that infect bacteria specifically. Because of their natural selectivity, the phages can be engineered to serve as both capture elements and signal amplifiers, especially for live cell detection. Finally, molecularly imprinted polymers (MIPs) or molecularly imprinted polymers, which have synthetic receptors with templated binding cavities with a shape and chemical resemblance to target molecules or microorganisms, are being harnessed for their robustness, reusability, and resilience to the often harsh processing environments encountered in the food matrices [80].

### Transduction Mechanisms

Once the pathogen is captured by the recognition element, the transducer generates the signal. The majority of studied platforms have focused on electrochemical biosensors, which measure changes in current, potential, or impedance upon target interaction [72]. Due to their compatibility with miniaturized formats and low power consumption, they hold great promise for portable and field-deployable detection kits. Optical biosensors rely on the variation of light properties such as fluorescence, absorbance, and surface plasmon resonance (SPR) to monitor binding events. surface-enhanced Raman scattering (SERS), an advanced technique, has reached a sensitivity level of a few cells and is increasingly integrated with nanomaterials for multiplexed detection [81, 82]. Piezoelectric biosensors, including quartz crystal microbalance systems, detect minute mass changes upon pathogen binding by measuring changes in oscillation frequency, providing label-free and highly sensitive detection. Colorimetric biosensors have become popular for their simplicity, low cost, and ease of visual readout; paper-based formats, especially, are in great demand in resource-poor settings. Most of these involve the use of gold nanoparticles, nanozymes, or other materials that change color visibly in response to the presence of bacteria [22, 83, 84].

### Performance Metrics: Sensitivity, Specificity, LOD, Response Time

Several important factors must be taken into account to assess how well a biosensor actually works. The first of these is ‘sensitivity’, which refers to its ability to detect low concentrations of the pathogen one is interested in. Most regulatory bodies will require a lower limit of detection (LoD) than 10 CFU per mL or g for many harmful pathogens. Another important factor, specificity, is the ability of the sensor to detect only what it is supposed to - i.e. the target pathogen among many other non harmful or ‘background’ microorganisms in a complex food mixture [85]. The limit of detection (LOD) quantifies the lowest concentration at which the biosensor can reliably identify the pathogen. With nanomaterials and signal amplification strategies, modern biosensors can achieve LODs in the range of 1–10 CFU/mL, rivaling or surpassing traditional molecular methods. The response time is another key parameter to consider; usually, the most recent biosensors will provide results in 10–30 min, representing a significant benefit over culture-based methods taking days. What about portability, simplicity of use, and dependence/stability/invariance of operation in different environments? All of these are important case studies in realistic food safety monitoring situations, at critical control points e.g. processing, transportation, retail, consumer homes, etc. [86, 87].

#### Distinguishing Viable From Non-viable Targets in Nucleic-acid Biosensing

A well recognised drawback of nucleic-acid-based biosensors is their inability in isolation to distinguish live and dead, as DNA may persist post cellular death. To address this challenge, several biosensing strategies have been developed to preferentially detect viable organisms. Some frequently used biosensors employ RNA-targeted detection, particularly mRNA or rRNA, which decays rapidly after cell death and, therefore, can be considered an indicator of metabolic activity. Biosensors targeting bacterial rRNA or virulence-associated mRNA transcripts have demonstrated improved correlation with cell viability compared to DNA-based assays [88–91] .

Another interesting approach makes use of viability dyes (such as propidium monoazide (PMA) and ethidium monoazide (EMA), which penetrate into and partially inhibit the amplification or detection of DNA in membrane-compromised (that is, dead) cells. Coupled with an electrochemical or optical biosensor, PMA/EMA pretreatment enables selective viability based detection of viable pathogens detected in complex food matrices. Metabolic activity-based biosensing offers an alternative viability marker by simply waiting for signals associated with respiration (strongly electrochemical ones), display of enzyme activity, or even ATP release or redox activity of those redox-active nanoparticle nanozymes only by live cells. These approaches are suitable for rapid screening in the absence of nucleic acid amplification [92–95]. Moreover, bacteriophage-based biosensors assay only viable cells, since phages infect and grow only in metabolically active bacteria. Phage-induction and subsequent detection methods have been used for the live-cell specific detection of E. coli, Salmonella and Listeria providing a biologically selective route to viability assessment. Overall, these approaches emphasise that while biosensors certainly identify nucleic acids originating from both live and dead cells, viability discrimination could be realised through appropriate target selection, as well as pretreatment and signal transduction design, for a more actionable information from biosensing platforms intended for food safety [96, 97].

#### Multiplex Detection Challenges and Cross-reactivity

Multiplex detection of foodborne pathogens poses further difficulties, with challenges arising such as bioreceptor cross-reactivity, signal overlap, and matrix interference. This is especially the case when structurally similar pathogenic microorganisms are analysed from heterogeneous mixtures of complex food matrices. For example, antibodies or aptamers may still bind partially with non-target organisms, and the optical signal produced by several different analytes may overlap each other, inhibiting quantification. As a solution to these problems, it has become increasingly common to implement biosensing platforms which use spatially separated sensing zones, orthogonal biorecognition elements (such as antibody–aptamer combinations), and/or ratiometric or multi-frequency signal transduction schemes. While microfluidic compartmentalization also reduces cross-talk by conducting reactions in separate channels or droplets. Importantly, AI and ML-based signal can be used to resolve multiplexed to-from signals by learning to identify fine signals indicative of particular pathogens, in the presence of noise and/or partial cross-reactivity. Such data-driven techniques can greatly enhance specificity and robustness, especially in food matrixes in which perfect biochemical selectivity is not likely to be obtained [98–100].

## Emerging Materials and Functional Elements

The emergence of biosensing platforms for the detection of foodborne pathogens is driven significantly by the demand for new materials and functional elements that improve the sensitivity, stability, specificity, and adaptability of the sensors. While these new materials improve the limits of detection and analytical performance of the biosensors, they also deliver benefits related to miniaturization, multiplexing, and cost-effective manufacture, which are important traits for food safety monitoring across the various steps of the food supply chain. In recent years, researchers have leveraged advances in nanotechnology, smart polymers, synthetic biology, and enzyme mimics to engineer highly responsive, robust, and multifunctional biosensing platforms tailored for bacterial detection in complex food matrices.

### Nanomaterials for Signal Amplification

**Fig. 2:**
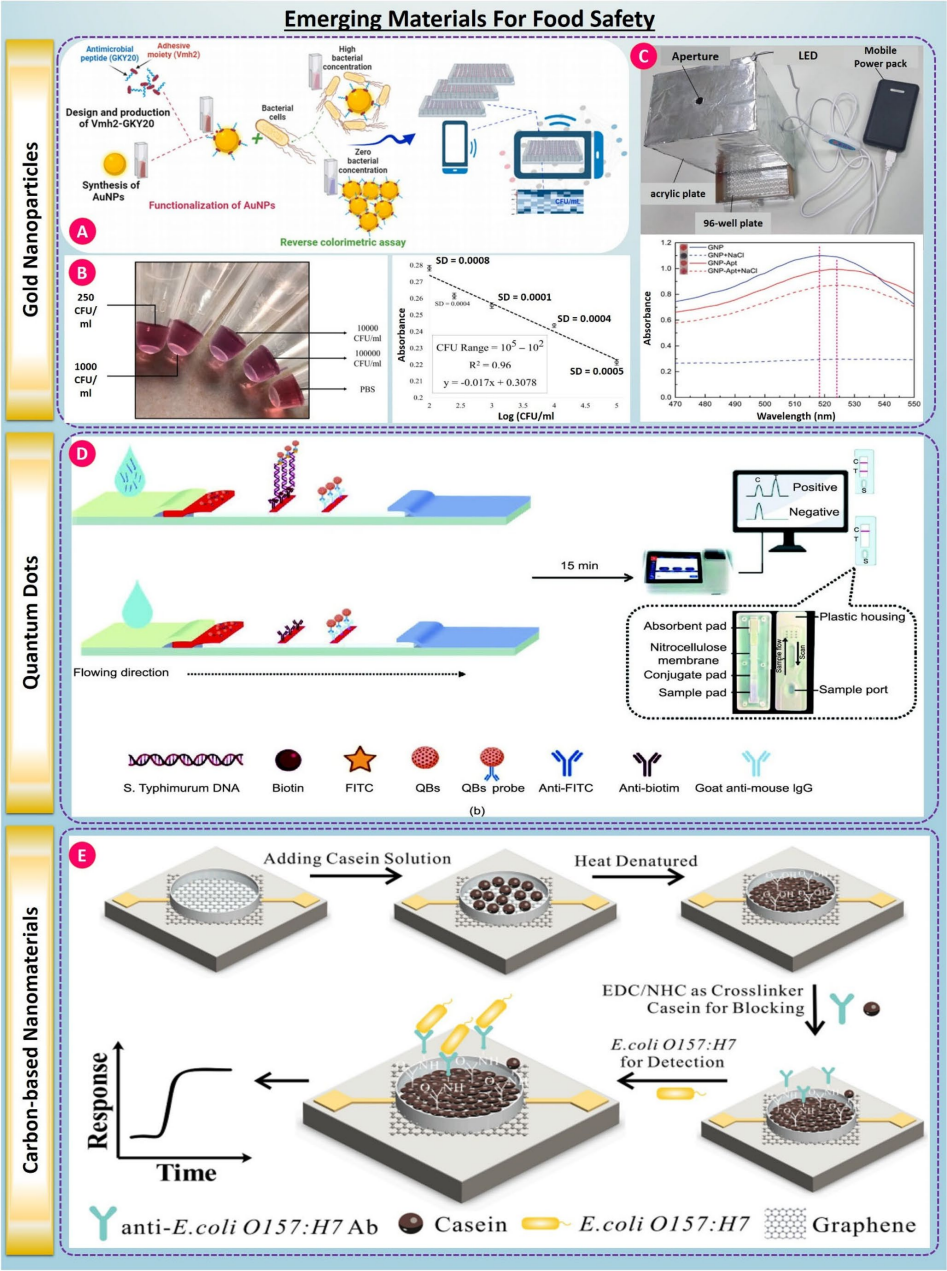
(**A**) Functionalized AuNPs enable a reverse colorimetric aggregation assay for bacterial detection, with quantification via smartphone/microplate readout, taken from [106] with the permission of Elsevier. (**B**) Test-tube color changes across serial CFU levels and a log(CFU/mL) calibration showing strong linearity (illustrative R² ~0.96) and broad dynamic range, taken from [105] with the permission of Elsevier. (**C**)Low-cost light enclosure for consistent illumination and spectral capture; representative absorbance spectra demonstrate aggregation-induced peak shifts/intensity changes taken from [107] with the permission of Elsevier. (**D**) QDs lateral-flow test: Biotin/FITC-labeled QD probes capture Salmonella (S. Typhimurium) DNA on nitrocellulose; anti-biotin/anti-FITC lines yield a fluorescent yes/no result in ~ 15 min, taken from [108] with the permission of Elsevier. (**E**) Carbon-based nanomaterials (graphene): Heat-denatured casein cross-linked on graphene electrodes blocks nonspecific sites; anti-E. coli O157:H7 antibodies enable target binding. Right: graphene field-effect transistor (GFET) with Au source–drain and liquid gate for label-free electrical detection, taken from [114] with the permission of Elsevier

Nanomaterials are among the most innovative materials that have been applied to biosensor design as a result of their unique physicochemical properties such as high surface area-to-volume ratio, enhanced conductivity, and tunable surface functionalities. Gold nanoparticles (AuNPs) have often been used to amplify biosensor signals in colorimetric and electrochemical biosensors [101–103]. Their high surface plasmon resonance effect enables highly sensitive detection using an optical signal that is often visible to the naked eye [104]. For example, AuNP-based colorimetric biosensors have been developed for the rapid visual detection of E. coli O157:H7 in milk and water samples, where target-induced nanoparticle aggregation results in a distinct color change (see Fig. 2(A-C)) [105–107]. Similarly, quantum dots (QDs), semiconductor nanocrystals with size-tunable fluorescence, are increasingly employed for fluorescent biosensors due to their high brightness and resistance to photobleaching. These have enabled multiplexed Listeria monocytogenes and Salmonella [108] (see Fig. 2D) serovars detection in complex samples using aptamer-conjugated platforms [109, 110].

Carbon-based nanomaterials, particularly graphene oxide (GO) and reduced graphene oxide (rGO), and carbon nanotubes (CNTs), have gained considerable interest for their outstanding ability to transfer electrons and for their large loading capacity of biorecognition elements [111–113].

Graphene-based field-effect transistors (FETs) tailored with aptamers or antibodies have successfully displayed the capability to detect pathogens in real-time with sensitivity as low as sub-10 CFU/mL (see Fig. 2D) [114, 115]. In addition, MXenes, a new class of 2D transition metal carbides/nitrides, are producing high performance materials because of their hydrophilicity, metallic conductivity and many surface terminations, MXene-based electrochemical biosensors are showing promise for detection of Salmonella in meat samples, producing rapid and stable signal output, and minimal sample preparation [95].

### Smart Polymers and Hydrogels

**Fig. 3:**
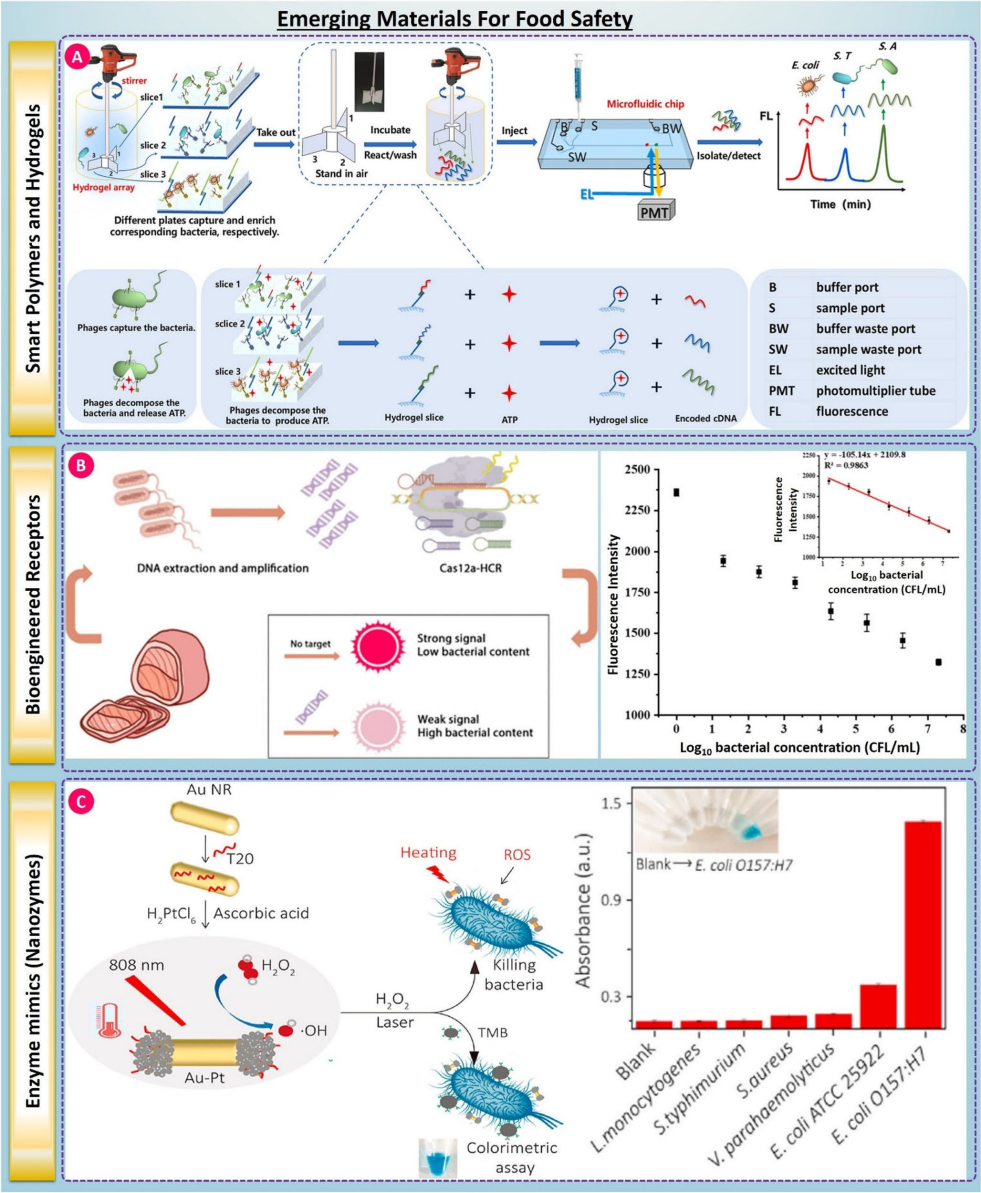
(**A**) Smart polymers & hydrogels: A bacteriophage-functionalized hydrogel array captures different bacteria on separate slices; phage lysis releases ATP, which is routed to a microfluidic chip for fluorescence readout. Representative multiplex traces show distinct signals for E. coli, S. Typhimurium, and S. aureus, taken from [118] with the permission of Elsevier. (B) Bioengineered receptors: CRISPR/Cas12a coupled with hybridization chain reaction (Cas12a–HCR) converts amplified pathogen DNA into a measurable signal, taken from [123] with the permission of ACS. (**C**) Enzyme-mimic nanozymes: Plasmonic/metal nanozyme composites combine photothermal heating (808 nm) with peroxidase-like catalysis to kill/enrich bacteria and amplify colorimetric assays. Magnetic separation enables rapid and selective detection, illustrated here for E. coli O157:H7 against common interferents, taken from [130] with the permission of Elsevier

Furthermore, the inclusion of stimuli-responsive smart polymers and hydrogels has added a dynamic element to biosensor design objectives along with nanomaterials [116, 117]. These polymers can respond to environmental conditions such as pH and temperature, or target binding events that permit physical or chemical changes. For example, thermoresponsive hydrogels with specific aptamers could swell or shrink after pathogen recognition depending on the water uptake as it recognizes the target or unbound pathogen, allowing for modulation of the fluorescence or electrochemical signal (see Fig. 3A) [118]. These platforms enable real-time, reversible, and self-regulated sensing useful in smart packaging and cold-chain monitoring systems. Molecularly imprinted hydrogels have also been employed to enhance selectivity and reusability, particularly for Listeria detection in ready-to-eat foods [119, 120].

### Bioengineered Receptors

Recent advances in synthetic biology and molecular engineering have expanded the toolbox of recognition elements available to biosensors, including CRISPR/Cas-based detection systems, which offer programmable sequence-level specificity, built-in signal amplification, and compatibility with optical, electrochemical and microfluidics transduction formats. The identification of target nucleic acid sequences occurs with the use of guide RNAs to aid in recognition, followed by the cleavage of targeted nucleic acids via collateral cleavage activity by Cas enzymes (i.e., Cas12a or Cas13a) which generate measurable reporter signals. This mechanism enables ultrasensitive detection of foodborne pathogens, including *Escherichia coli* and *Salmonella*, often reaching copy-level sensitivity without thermal cycling, thereby supporting rapid and portable diagnostic configurations [121, 122].

CRISPR-enabled biosensors go beyond just being sensitive and also provide multiple advantages over other assay types in terms of programmability, multiplexing, and compatibility with decentralized or point-of-care use. However, many of the same analytical and translational limitations that are found in laboratory settings will also apply to food safety applications in practice. Nonspecific collateral cleavage may elevate background signals, while matrix-derived inhibitors, such as proteins, lipids, salts, and polyphenols, can suppress amplification efficiency or Cas activity, reducing sensitivity and reproducibility in complex samples [123]. In addition, many CRISPR assays are still threshold based rather than fully quantitative in nature, making accurate pathogen load estimation impractical in comparison to calibrated molecular diagnostics. Variability in enzyme stability, coupling amplification, and preprocessing present added hurdles to inter-laboratory standardization and regulatory validation. Dealing with these limitations through matrix-tolerant chemistries, signal-normalization strategies, integrated microfluidic pretreatment, and standardized validation workflows will be an important part of translation. Overall, bioengineered receptor technologies, and especially CRISPR-enabled systems, represent a potentially transformative direction in biosensing that integrates molecular precision with field-deployable food-safety diagnostics [124, 125].

### Enzyme Mimics (Nanozymes)

In parallel, enzyme mimics known as nanozymes are gaining prominence for their catalytic activity and stability under harsh conditions, which overcome many limitations of natural enzymes [126, 127]. Nanozymes such as iron oxide nanoparticles, cerium oxide, and carbon-based composites exhibit peroxidase-like activity and have been integrated into colorimetric and electrochemical biosensors [128, 129]. For example, Au@Pt nanozymes have been used to catalyze H₂O₂ decomposition in the presence of bacterial cells, enabling the detection of E. coli in less than 20 (see Fig. 2C) minutes through amplified colorimetric signals [130]. These nanozyme-enhanced biosensors are particularly valuable in detecting bacteria in opaque or colored food matrices where optical clarity is compromised [131].

Combining these new materials to support biosensor performance will lead to next-generation biosensing platforms that can be adaptable, multifunctional, and operable in a wide range of food contexts. A number of these materials are compatible with high-throughput manufacturing (printing or coating), making them suitable for disposable and point-of-care diagnostic kits. Their potential for use with flexible substrates and miniaturized electronics within biosensors makes them promising candidates for developing wearable sensors, intelligent food packaging and mobile-phone readers [132].

In conclusion, biosensors for the detection of foodborne pathogens have been re-engineered using nanomaterials, smart polymers, CRISPR-based biorecognition elements, and nanozymes, which has created biosensors with improved sensitivity, reduced assay times, increased specificity, and made more versatile biosensing platforms. As research continues to explore novel combinations and material hybridizations, future biosensors will likely exhibit even greater functionality, adaptability, and field-readiness, paving the way toward robust, real-time food safety monitoring systems.

## Integration with Advanced Technologies

As biosensing approaches for foodborne pathogens become more ubiquitous, integrating existing and emerging digital, microfabrication and analytical approaches become imperative. These enable us to push the functional boundaries of what biosensors can accomplish whilst harvesting the advantages of portability and multiplexing, and easy access and use, logical decisions in realtime. In this section, we present three enabling technology groups critical for driving advances in biosensing. First, we discuss microfluidics and lab-on-a-chip devices, detailing how such compact, integrated systems streamline sample handling, analysis, and decision-making in one platform. Then we address sensing approaches made possible by smartphones and IoT capabilities, emphasizing how mobile and connected devices extend reach, enable real-time data capture, and facilitate remote monitoring. We conclude with an examination of biosensing enabled by artificial intelligence and machine learning, showing how advanced analytics can enhance interpretation, pattern recognition, and predictive insight. Each enabler thus contributes to the development of next-generation biosensing platforms, and each subsection offers deeper insights into bridging current gaps in sensitivity, specificity, and usability en route to intelligent, decentralized, and predictive food safety diagnostics.

### Microfluidics and Lab-on-a-chip Devices for Food Safety

The coupling of microfluidics and lab-on-a-chip technology can be seen in bacterial biosensing for food safety application through compact, autonomous, and rapid diagnostics. Here, fluid handling happens at microliter to nanoliter scale, which can reduce the reagents and detection time, and increase assay sensitivity. These systems can perform a variety of operations, such as filtration, enrichment, mixing, reaction and detection of samples all on a single chip, reducing the chances of contamination and human error. This requirement for sample-in-answer-out, efficiently operated even in decoupled or low-resource scenarios, is particularly significant for foodborne pathogens, such as *E. coli*, *Salmonella*, and *Listeria* monocytogenes [133, 134]. This paradigm shift from centralized laboratory analysis to portable, real-time field diagnostics aligns well with global food safety frameworks demanding rapid, sensitive, and user-friendly solutions.

**Fig. 4:**
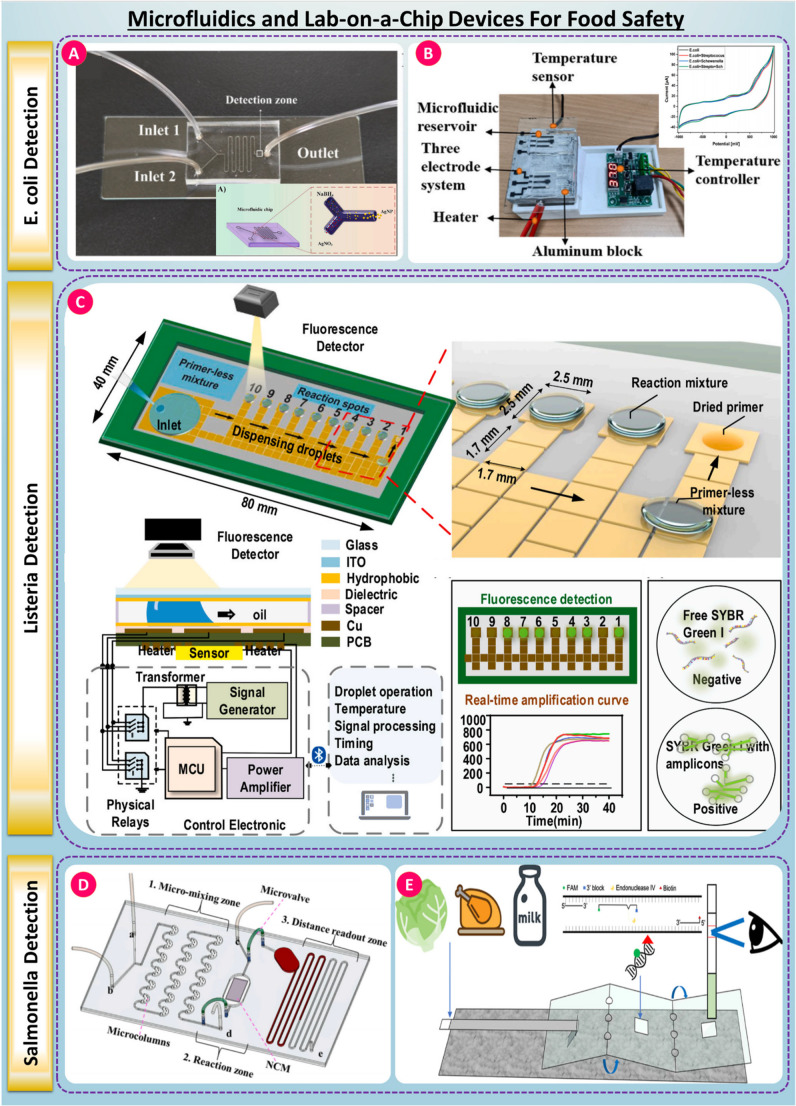
(**A**) Microfluidic chip designed with dual inlets and an integrated detection zone, enables precise sample handling for efficient *E. coli* detection, taken from [135] with the permission of Elsevier. (**B**) An electrochemical system with a three-electrode setup and temperature control facilitates sensitive *E. coli* detection under regulated thermal conditions, taken from [136] with the permission of Elsevier. (**C**) Digital microfluidic device for droplet-based *Listeria* detection, uses isothermal amplification and fluorescence readout for rapid analysis, taken from [139] with the permission of Elsevier. (D) A microfluidic platform with mixing, reaction, and readout zones supports integrated processing and detection of Salmonella, taken from [140] with the permission of Elsevier. (**E**) Nucleic acid-based lateral flow assay for Salmonella identification provides visual output through fluorescence-labeled probe detection, taken from [141] with the permission of ACS.

For addressing particular challenges, different novel microfluidic biosensors have been developed for bacterial detection in diverse food matrices. One of the microfluidic-SERS platforms for E. coli detection, developed by Heera Jayan et al. shows in-situ synthesis of silver nanoparticles using a Y-junction serpentine channel, as illustrated in Fig. 4A. Due to specific targeting by aptamer-guided nanoparticle synthesis, the biosensor was able to achieve a detection limit of 1.1 CFU/mL and performed well in lettuce samples, one of the most important advances of Raman-based bacterial biosensing [135]. Such highly sensitive microfluidic–SERS architectures are directly translatable from a healthcare perspective to clinical pathogen screening, where the presence of low bacterial loads in biological fluids needs to be identified as quickly as possible for early diagnosis and infection control. In another case, [136] introduced a microfluidic electrochemical chip enhanced with graphitized mesoporous carbon electrodes that enabled real-time, incubator-free culturing and electrochemical monitoring of E. coli growth, as shown in Fig. 4 (B). This system achieved an outstanding LOD of 0.35 CFU/mL and was successfully validated in mango juice. Along with these, Puiu and Bala detailed a survey of microfluidic platforms, featuring portable systems developing SPR, SERS and lateral flow for point-of-care food diagnostics, demonstrating further flexibility of microfluidics in low-resource or in-field applications [137].

[138] introduced a paper-based colorimetric µPAD for monitoring surface contamination in detecting Listeria. Sensitive detection of 10² CFU/coupon of L. monocytogenes after enrichment was achieved, particularly on the food contact surface made of stainless steel and plastic. Meanwhile, a digital microfluidic chip that involved the integration of loop-mediated isothermal amplification for the multiplexed detection of Listeria, E. coli, and Salmonella was introduced by [139] as illustrated in Fig. 4 (C). This chip processed 10 targets simultaneously with an LOD of 10² CFU/mL and completed analysis within 50 min.

[140] proposed aptasensor distance-based microfluidic capable of providing a visual readout without requiring power, reaching a LOD of 3.7 × 10¹ CFU/mL through gasmediated displacement gold nanoparticles, Fig. 4 (D). As well, [142] reported a high-throughput microfluidic chip based on recombinase-aided amplification for detecting Salmonella serogroups, allowing multiplexed identification of five clinically important serogroups with a LOD of 10¹ CFU/mL in only 15–40 min. Although demonstrated primarily for food matrices, these systems mirror nucleic-acid–based microfluidic platforms used in clinical microbiology, reinforcing their relevance for rapid infection screening and public-health surveillance. Finally, Chen et al. described an origami-like polyethersulfone microfluidic chip incorporating a paper-based DNA extraction step, RPA amplification, and lateral flow readout in a completely integrated, sample-to-answer detection of Salmonella enterica in milk and lettuce with LODs < 10 CFU/g, approaching the ASSURED criteria for field deployability in a scheme depicted in (E) of Fig. 4 [141]. Concomitantly, this system meets several ASSURED criteria, which include being Affordable, Sensitive, Specific, User-friendly, Rapid, Equipment-free, and Deliverable, which positions it not only as a food safety tool but also potentially as a point-of-care diagnostic platform for low-resource health settings.

Collectively, all the case studies mentioned clearly reveal that microfluidic and lab-on-a-chip biosensors developed for the detection of foodborne pathogens intrinsically bear strong relevance to healthcare. Indeed, portability, rapid turnarounds, multiplexing potential, isothermal amplification compatibility, and smartphone readout make them suited for point-of-care diagnostics, outbreak investigation, and public-health monitoring. The capability for early detection of pathogenic bacteria before widespread exposure or clinical manifestation gives microfluidic biosensing platforms a critical dual role: enhancing food safety and supporting preventive health care and infectious disease control.

### Smartphone and IoT-Enabled Sensing Platforms for Food Safety

With their embedded cameras, processors, connectivity, and ease of use, smartphones increasingly join biosensing platforms for on-site monitoring of food safety. Conventional immunoassays could thus be converted into portable, inexpensive, real-time diagnostics for use in industrial applications and even in remote areas. Paired with cloud analytics, ML algorithms, and Bluetooth or app-based readouts, smartphones can enable rapid quantification, remote access, and data sharing amongst stakeholders in the food safety ecosystem [62]. Biosensors combined with IoT architecture extend their capability from mere detection to predictive analytics, blockchain-based traceability, early warning system along the farm-to-fork chain. This section highlights a range of smartphone-enabled sensing strategies developed for E. coli, Salmonella spp., and Listeria monocytogenes, emphasizing their biosensing principles, analytical performance, and future potential [145]. 

**Fig. 5:**
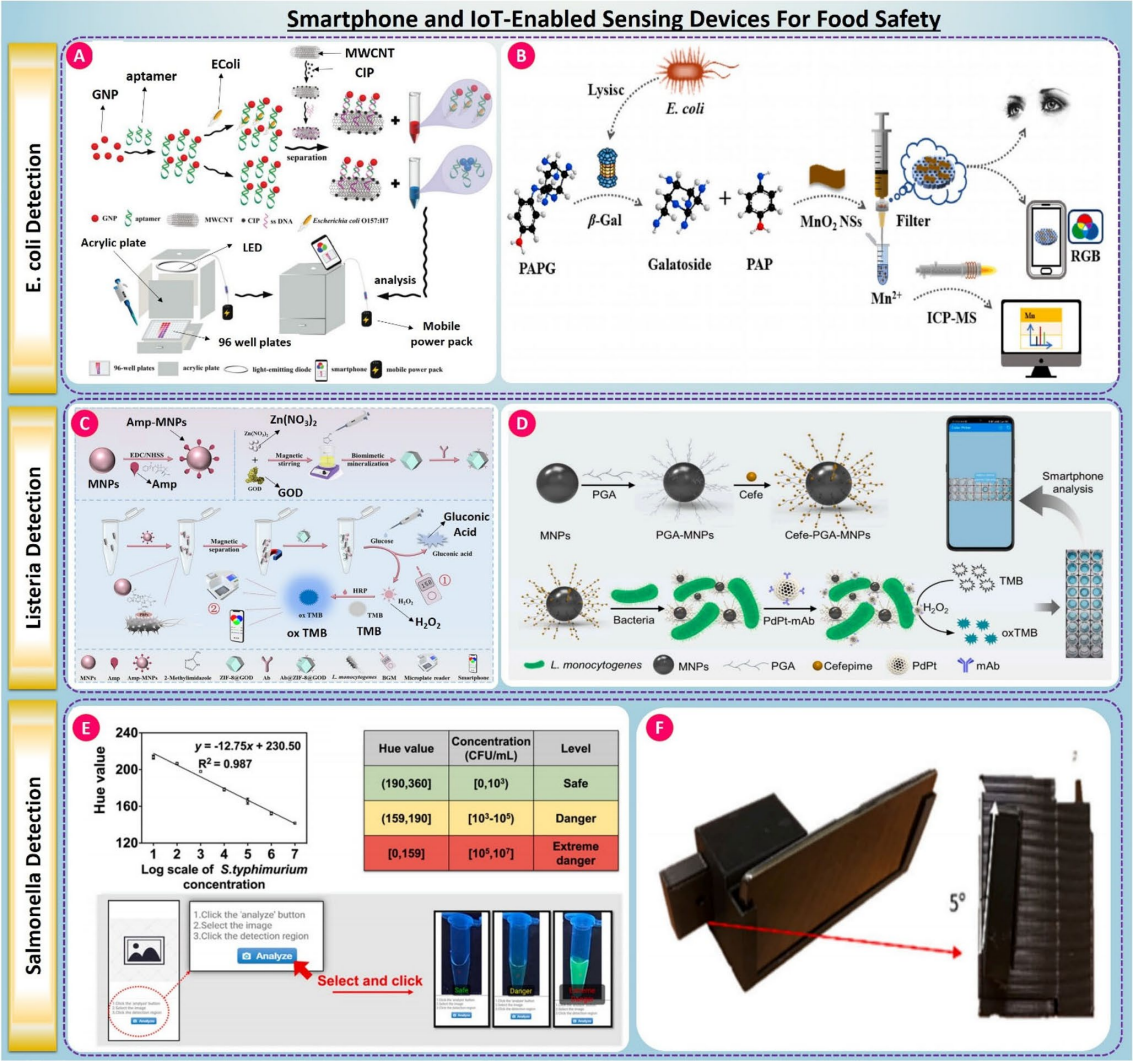
(**A**) Smartphone-enabled aptamer-magnetic nanocomposite sensor for E. coli O157:H7 detection, taken from [146] with the permission of Elsevier. (**B**) β-Galactosidase-based colorimetric E. coli detection using smartphone RGB and ICP-MS, taken from [147] with the permission of Elsevier. (**C**) Smartphone-integrated immunomagnetic fluorescence assay for detecting Listeria monocytogenes, taken from [149] with the permission of Elsevier. (**D**) Magnetic nanoprobe and TMB-based smartphone assay for Listeria detection, taken from [151] with the permission of Elsevier. (**E**) Hue-based smartphone colorimetric system for Salmonella (S. Typhimurium) quantification, taken from [143] with the permission of Elsevier. (**F**) 3D-printed smartphone holder for standardized imaging in food safety assays, taken from [152] with the permission of Elsevier

[146] proposed an interesting smartphone-based colorimetric aptasensor for the determination of E. coli based on mixed functionalized gold nanoparticles together with magnetized multi-walled carbon nanotubes (Fig. 5(A)). The authors sensed E. coli at a limit of 524 CFU/mL under 1 h in milk and facilitated its separation and acquisition of visual signal by a customized mobile device. Besides food safety, this platform serves as a model point-of-care diagnostic for healthcare applications where detecting the presence of bacterial contamination in water or biological fluids is critical to the early assessment of infection risks and the prevention of disease. Extending this concept, [147] Further introduced a dual-mode biosensor combining smartphone-readable colorimetry and ICP-MS detection, leveraging β-galactosidase activity to convert MnO₂ nanosheets into Mn²⁺ ions, with LODs of 5.6 × 10³ CFU/mL (colorimetric) and 35 CFU/mL (ICP-MS) in under 1 h for diverse matrices, as shown in Fig. 5 (B). In terms of field integration, both systems demonstrated effective signal acquisition via mobile phone cameras, real-time decision-making, and compatibility with visually opaque food matrices. In another study, Xing et al. advanced the field with a microfluidic FRET-based smartphone detector capable of simultaneous detection of four foodborne pathogens’ ssDNA, including E. coli, achieving multiplex LODs down to 0.17 nM in under 5 min. The system supports real-time, wash-free detection, illustrating high-throughput IoT-readiness for food safety diagnostics [148]. This ultra-fast, multiplex smartphone readouts can translate to emergency and field triage for infectious gastroenteritis, reducing delays to isolation and targeted care.For Listeria monocytogenes, Bai et al. presented a dual-mode biosensor combining smartphone colorimetry and glucometer-based glucose oxidase readout, with 10¹ CFU/mL sensitivity in lake water and juice [149], as shown in Fig. 5 (C). The reuse of glucometer infrastructure—widely deployed in healthcare for glucose monitoring, underscores the translational potential of this approach for infectious disease diagnostics in low-resource clinical settings. For Salmonella detection, Mahari et al. fabricated an immunochromatographic assay integrated with the ColorGrab smartphone application, achieving the detection in spiked fecal and food samples at LODs of 10³-10⁴ CFU/mL across different Salmonella strains in 10 min. This configuration presented excellent usability when kept under cold and room temperature storage, besides which field deployment was also facilitated by the use of smartphone-mediated color interpretation. The rapid time-to-result and ease of use highlight the platform’s suitability for both food safety inspections and community-level healthcare screening [150].

In another study, [151] designed a magnetic nanozyme-based smartphone colorimetric assay (see Fig. 5 (D)) capable of capturing over 90% of Listeria cells in real samples and detecting as low as 31 CFU/mL. Finally, Liu et al. utilized SYTO9 staining with smartphone imaging and LAMP amplification, achieving detection of Listeria at 6 copies/µL through image-based fluorescence analysis [153]. Each of these systems showed seamless smartphone integration, with automated signal quantification, portability, and suitability for environmental and food sector applications. Their modular nature suggests future integration with cloud dashboards, enhancing traceability and predictive risk assessment.

Complementarily, [143] proposed a smartphone-assisted fluorescence sensor based on magnetic COF-AuNPs with aptamer targeting and reached 4 CFU/mL sensitivity for S. typhimurium in 33 min, as seen in Fig. 5E. Smartphone imaging enabled the real-time readout of fluorescence quenching that coupled lab-grade sensitivity to portable systems. [152] also proposed a lateral flow assay interpreted using a smartphone that employed ML algorithms and improved the accuracy of classification from fuzzy test lines through SVM and KNN classifiers by 95.6% and reduced visual inspection errors in cell concentrations, as shown in Fig. 5(F). This approach addresses one of the main shortcomings of visual diagnostics, which is subjective interpretation; and closely reflects healthcare diagnostics where algorithm-assisted decision support is increasingly deployed to reduce operator bias and enhance diagnostic confidence.

Finally, smartphones and IoT-enabled biosensing platforms publish at a point where the safety monitoring of food converges. With the portability, connectivity, response time, and ability to complement with AI-driven analytics, these sensing platforms are ideal for point-of-care testing, and testing surveillance of public health and prevention of outbreaks. Not only do these make food supply chains more safe, they also promote proactive healthcare and the control of infectious diseases by way of early pathogen detection at the interface between food systems and human exposure.

### AI/ML-assisted Biosensing Platforms for Food Safety

The application of AI-ML to the biosensing platforms revealed a new era of foodborne pathogen detection with improved accuracy, faster, and real-time adaptability. Conventional biosensors have performed fairly well in what they do but are often hindered by variability, food matrix complexities, and subjective interpretation, especially in field settings. Presently, AI/ML algorithms are integrated with optical, spectroscopic, and nanomaterial biosensors to overcome limitations by providing intelligent pattern recognition, better signal quantification, and data-driven diagnostics [154, 155]. The implementation of these advanced smart biosensing technologies enables organizations to develop more efficient food safety programs. This approach utilizes decentralized testing methods, advanced predictive monitoring capabilities, and integrated automated decision-support systems, thereby representing substantial progress toward achieving the goals associated with industry four point zero and the advancement of Smart Healthcare objectives.

**Fig. 6:**
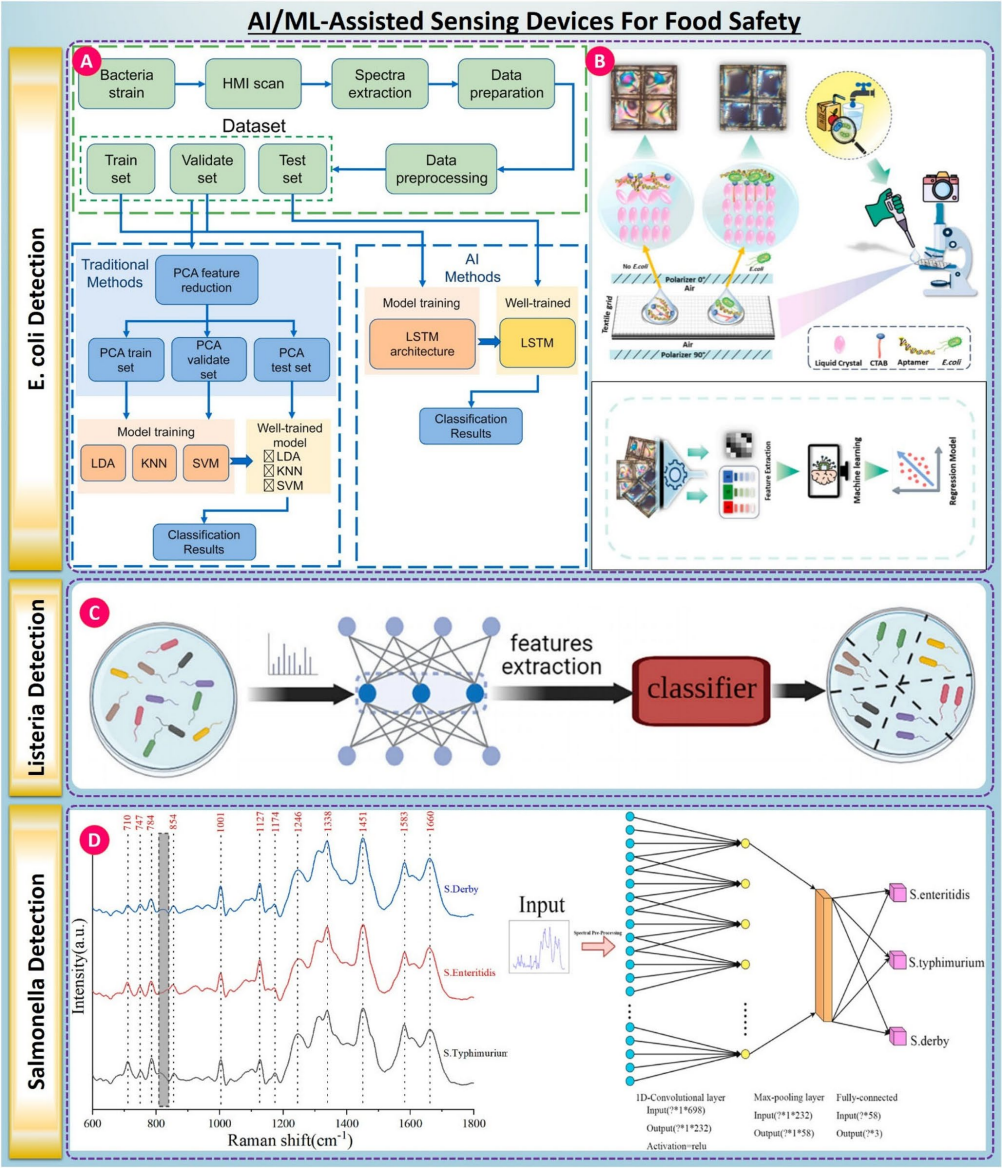
(**A**) ML workflow for E. coli detection using spectral data and LSTM classifiers, taken from [156] with the permission of Elsevier. (**B**) Polarized optical sensing system with AI-assisted image classification for E. coli detection, taken from [157] with the permission of Elsevier. (**C**) Deep learning-based feature extraction and classification model for Listeria identification, taken from [158] with the permission of Elsevier. (**D**) Raman spectroscopy combined with CNN-based deep learning for Salmonella serotype discrimination, taken from [159] with the permission of Elsevier

Recent case studies demonstrate the powerful synergy between biosensors and AI/ML techniques. In one study, Kang et al. developed an AI-assisted hyperspectral microscopic imaging system coupled with a long short-term memory network to identify five foodborne pathogens, including E. coli and Salmonella, at the single-cell level, as shown in Fig. 6 (A). The model achieved a classification accuracy of 92.9% using spectral data from the center region of cells, outperforming traditional PCA-LDA and SVM classifiers, thereby illustrating the capability of deep learning in capturing subtle spectral differences for rapid diagnostics [156]. Although demonstrated here for foodborne pathogens, the approach is directly relevant to healthcare diagnostics, where hyperspectral and AI-assisted imaging techniques are increasingly being applied to bring speed to pathogen identification from clinical samples and to reduce diagnostic turnaround time in microbiology laboratories. Building upon this, [157] presented a liquid crystal-based aptasensor that leverages polarized microscopy and ML algorithms (ANN, XGBoost) to detect E. coli in water and juice, as shown in Fig. 6 (B). The platform achieved detection limits of 6 CFU/mL with R² > 0.97, showing exceptional performance across sample types. A complementary approach by [160] used bacteriophage-based biosensors and CNNs trained on image datasets to recognize bacterial morphology changes, achieving 80–100% accuracy in real-world water and food matrices. Listeria-focused innovations include [161] work, which applied pan-genome features with majority-vote classifiers to predict L. monocytogenes virulence potential, obtaining F1 scores up to 0.88. This genomic-AI integration provides insights into pathogenicity rather than presence alone, a capability that is highly relevant for healthcare and public-health decision-making, where risk stratification informs intervention priorities. Similarly, [158] achieved 100% classification accuracy of six Listeria species using MALDI-TOF mass spectrometry combined with denoising autoencoders and SVMs, as shown in Fig. 6 (C), while [162] introduced a deep learning-metabolomics hybrid platform that distinguished L. monocytogenes with CNN-based accuracies exceeding 96%.

For Salmonella detection, Yan et al. employed single-cell Raman spectroscopy with kernel PCA and hierarchical decision tree classifiers to reach > 95% accuracy in pathogen serotyping [163], while Sun et al. optimized spectral preprocessing and CNN models to identify three major Salmonella serovars with > 98.5% accuracy, as shown in Fig. 6 (D) [159]. In another example, [164] utilized whole genome sequencing data and multiple ML classifiers, identifying stress-response genes associated with Salmonella adaptation in poultry environments, achieving AUROC values up to 0.904. While developed in a food-production context, such predictive genomic-AI models are directly applicable to healthcare epidemiology, where understanding pathogen adaptation and transmission dynamics is critical for outbreak prevention and antimicrobial stewardship.

In a nutshell, the AI/ML-assisted biosensing platforms represent an important junction of biosensor engineering, data science, and digital health. By improving sensitivity, specificity, interpretability, and operational robustness, AI-powered biosensors go beyond food safety monitoring into directly supporting healthcare diagnostics, public-health surveillance, and outbreak response. Capable of translating raw biosensing data into clinically relevant insight, they are well positioned to enable intelligent pathogen detection systems of the next generation at the food–health interface. To clarify such practical choices in this space we present a general comparison of certain key integrated approaches, across modalities of electrochemical, optical, microfluidic, AI/ML-assisted, and smartphone/IoT-integrated platforms. Shown side by side, this schematic Table 3 comparison provides some sense of functional tradeoffs per modality carefully balancing a range of criteria such as detection limits, complexity, cost per test, and appropriateness for use in the field.

**Table 3:** Comparative analysis of biosensing modalities

Parameter	Electrochemical Biosensors	Optical Biosensors	Smartphone/IoT-Integrated
Detection mechanism	Redox-based current or impedance via pathogen interaction	Fluorescence quenching, Raman scattering, SPR	Visual/colorimetric readout; fluorescence imaging via phone camera
Typical LOD (representative examples)	~ 1–10 CFU/mL in milk and juice using aptamer electrodes	~ 1 CFU/mL for Listeria via QD fluorescence	31–524 CFU/mL depending on imaging mode (colorimetric vs. ICP-MS)
Assay / response time (excluding enrichment)	~ 15–30 min	30–60 min (assay/optics dependent)	~ 10–30 min (real-time if colorimetric)
Representative sample matrices demonstrated (examples in this review)	Milk; juice; validated in milk-processing contexts	Juices/complex samples (optical background and matrix effects are relevant)	Milk; lake water; juice; spiked fecal + food samples
Key matrix interference / inhibitors	Impedance affected by lipids and proteins	Fluorescence quenched by polyphenols in juices	Color shifts can be masked by pigments or fat
Multiplexing potential	Dual/tri-analyte setups via interdigitated electrodes	Up to 4–6 targets using SERS, FRET, or wavelength-multiplexed detection	Usually single-analyte; some app-based switchable recognition schemes
Quantification reliability (practical note)	Typically quantitative with calibration; matrix drift possible	Quantitative with calibration; optical background can impact SNR	Often semi-quantitative; quantitative with app normalization and imaging control
Enrichment dependence (reporting note)	Case-dependent; times listed exclude enrichment if used	Case-dependent; times listed exclude enrichment if used	Case-dependent; times listed exclude enrichment if used
Portability	Handheld potentiostat with screen-printed electrodes	Often bench-top with optics and laser sources	Fully portable; smartphone + disposable strips or µPADs
Complexity of operation	Sample preparation + 2–3 step measurement	Light alignment, calibration, and trained handling	One-click app readout; minimal training
Cost per test	$0.5–2 with printed electrodes and reusable reader	$10–30/test due to fluorescent labels or optics	<$1/test with paper-based format + mobile app
Readout requirement	Potentiostat (portable or USB-based)	Spectrometer or fluorescence microscope	Smartphone camera, flashlight, or glucometer
Field suitability	Validated for on-site testing in milk processing units	Often limited to lab setups or controlled packaging facilities	Highly field-ready; used in cold chains and retail outlets

### Commercial Biosensor Platforms and Rapid Test kits (Current Landscape)

Beyond academic biosensor prototypes, the food industry uses a large number of commercially validated rapid testing platforms for screening and confirmation. These types of systems are all enriched-assisted LFIA screening kits or nucleic-acid amplification workflows (either RT-PCR or isothermal amplification) with automated readout. Table 4 presents representative commercial platforms, summarising the target scope, workflow characteristics, validation position, and practical strengths/limitations to provide context to the opportunity area for next-generation biosensors.

**Table 4:** Representative commercial platforms/kits for foodborne pathogen detection

Platform / Company	Reveal^®^ 2.0 (Neogen)	3 M™ Molecular Detection Assay 2 (Neogen)	SureTect™ PCR (Thermo Fisher)	iQ-Check (Bio-Rad)	foodproof^®^ Kits (Hygiena)	Assurance^®^ GDS (Merck)
Core Technology	Lateral Flow Immunoassay (LFIA)	Isothermal DNA amplification + bioluminescence	Real-time PCR	Real-time PCR	Real-time PCR	Immunomagnetic separation + real-time PCR
Target Pathogens	Salmonella spp.	Salmonella, Listeria	Salmonella spp., L. monocytogenes	Salmonella spp.	Salmonella, L. monocytogenes	Salmonella, Listeria
Workflow & Time to Result	Enrichment required; presumptive result ~ 24 h	Enrichment + automated real-time detection	Enrichment + PCR (~ 12–24 h total)	Single enrichment + PCR (~ 12–20 h)	Enrichment + PCR; LOD ≈ 1–10 cells/25 g	Enrichment + IMS + PCR
Sample Types	Meat, RTE foods, produce, environmental samples	Food matrices, environmental swabs	Food and production environments	Food, feed, environmental samples	Food and environmental samples	Complex food matrices
Validation Status	AOAC-RI PTM	Industry-standard validated system	AOAC / AFNOR validated	ISO / AOAC aligned	AOAC / MicroVal certified	Commercially validated
Key Strengths	Simple, low-cost, scalable screening	High throughput; standardized workflow	High specificity and sensitivity	Rapid confirmatory testing	Very high analytical sensitivity	Robust for inhibitor-rich samples
Key Limitations vs. Emerging Biosensors	Requires enrichment; qualitative; limited multiplexing	Instrument dependent; enrichment required	Lab-based; skilled personnel required	Requires PCR infrastructure	Higher cost; laboratory dependence	System-based; not field-deployable

## Key Challenges, Bottlenecks, and Possible Solutions

However rapid continues to be the development of biosensor design, materials, and technological integrations, there are still bottlenecks preventing the translation of these advances into real-life, large-scale food safety solutions. These bottlenecks prevent the performance of these sensors from working in complex food systems, and also impact the acceptance, reliability, scalability, and regulatory aspects of these systems. If the idea of biosensing platform technology is to become a reality in real life, it is imperative to address these bottlenecks, and determining exactly how to do so comprises part of the difficult technical challenge. The following subsections detail four major challenge areas, matrix interference and sample complexity, stability and reproducibility of biological elements, scalability, cost, and user-friendliness, and regulatory hurdles and standardization issues, while also highlighting innovative approaches and emerging solutions to overcome each of them.

### Sample Pretreatment and Enrichment Challenges

While some progress has undoubtedly been made in the improvement of the sensibility of biosensors and how integrated and streamlined they are, the processes of prep-treating and enriching samples for otherwise promising biosensors to be able to detect foodborne pathogens remains one of the most significant barriers to their eventual implementation for our various bioanalytical needs. The food matrix is inherently very complicated, consisting of all sorts of other fats, proteins, other carbs, phenol compounds, salts, and organisms that constitute the makeup of the native flora that all may interfere with a biosensor’s ability to pick out its target and signify it through its signalling pathway. Additionally, pathogens (such as Escherichia coli, Salmonella spp. and Listeria monocytogenes) are often found in food samples at concentrations that are at or below the limits of detection (e.g., < 10 CFU/g), necessitating the need for additional enrichment procedures to achieve compliance with regulatory detection thresholds [165–167].

Standard enrichment protocols, while effective, typically take 6–24 h or more, increasing time to result and jeopardizing the rapid response benefits of biosensing technologies. Selective enrichment media can also bias detection by preferring to enrich certain strains while suppressing others, particularly in stressed or injured cells, while manual pretreatment steps such as filtration, centrifugation and chemical lysis render the multiplexed assays too heterogeneous for on-site or decentralised testing. Matrix-derived inhibitors pose further hurdles for detection downstream. Lipids can foul electrochemical electrodes, polyphenols can quench fluorescence, and particulates can interfere with optical reads. Because of this biosensors showing good performance in buffers may exhibit diminished sensitivity and reproducibility in real food [168, 169]. This could occur via modifications to the immunomagnetic separation, microfluidics filtration, dielectrophoretic concentration and on-chip enrichment modules to reduce assay time and manual handling prior to detection. Nanozyme-assisted pretreatment and ratiometric sensing mitigate matrix effects, while AI-assisted signal correction aids in the control of true pathogen amongst noise (matrix effects). In its entirety this toolkit has the potential to turn sample pretreatment from its current bottleneck state into a seamlessly integrated part of the next generation of biosensing platforms [170].

### Matrix Interference and Sample Complexity

Matrix interference is one of the most critical and persistent challenges in biosensing for foodborne pathogen detection, particularly when dealing with heterogeneous food samples such as dairy, meat, leafy greens, or processed products [171]. The complexity arises from the coexistence of fats, proteins, polysaccharides, polyphenols, salts, and native microflora which can non-specifically bind to sensor surfaces, obscure target recognition events, or alter electrochemical/optical responses [172]. For instance, lipids in dairy products can interfere with electrochemical impedance in aptasensors, while polyphenols in plant-based foods can quench fluorescence in optical sensors. Furthermore, microbial detection in low-moisture foods or complex emulsions is challenged by uneven analyte distribution and the need for pre-enrichment, which not only delays detection but can also amplify matrix-associated artifacts [173, 174].

Crucially, these matrix-originating phenomena may yield false-positive and false-negative results in biosensing applications. As mentioned previously, false-negative scenarios may occur where target pathogens are present but evaded recognition due to epitope physical shielding, competitive adsorption of matrix components on sensing surfaces, intrusion into signal transduction pathways, or due to the inherent physiological state of bacteria impeding binding or signal generation. Conversely, false positives may arise due to non-specific adsorption of electroactive or optically active compounds that interfere with the sensor elements, cross-reactivity to background microflora, or detection of biomolecules (such as free DNA, endotoxins, etc.) from non-viable cells that aren’t implicated as a current contamination source. These error modes may be more pronounced under realistic food testing conditions compared to buffer-tests, resulting in a disconnect between lab-measured performance metrics and field reliability [86, 175, 176].

In order to circumvent the aforementioned constraints, technically advanced solutions have been introduced that preconceive work that moves beyond mere generic sample dilution or filtration techniques. One notable method involves utilizing microfluidic-based on-chip sample pretreatment modules that perform a real-time matrix separation step via size-exclusion, electrophoretic mobility, or dielectrophoretic principles [177]. For example, microfluidic chips that integrate acoustic wave sorting or electrokinetic enrichment have been used to separate and isolate bacterial cells from viscous matrices while providing the advantage of removing interfering solids/particulates. Another method that employs immunoseparation based on magnetic beads uses surface-modified magnetic beads that capture pathogens, thus providing clean extraction methods from food matrices with limited matrix carryover. Such physical separation steps directly reduce both false negatives (by enriching true targets) and false positives (by suppressing nonspecific background signals). This work can be bundled with AI signal deconvolution algorithms to distinguish where true pathogen-specific signals can be separated from the matrix-specific background noise [178–180].

In addition, nanozyme-assisted pretreatment is an emerging area of development in which catalytic nanomaterials (e.g., ceria, peroxidase-mimicking nanostructures) are used to degrade organic food residues or disrupt interfering biomolecules, while allowing for pathogen viability [180]. At the same time, ion-imprinted polymers and MIPs have been developed with specific binding to its target biomarkers, while repelling the contaminants from the matrix so that sensors that utilize MIPs as chemically stable interfaces can maintain selectivity in complex media. In terms of signal transduction, researchers are now exploring ratiometric sensing (e.g., dual-emission fluorescence or dual-frequency EIS) to correct for signal drift caused by matrix effects, enhancing detection reliability. When integrated with machine learning models trained on augmented datasets including matrix variability, these solutions significantly improve biosensor robustness across diverse food categories, offering a viable pathway toward real-world deployment [181].

### AI/ML-Related Challenges and Mitigation Strategies

AI/ML-enabled biosensing for foodborne pathogens is promising but currently incurs several limitations that must be explicitly recognized for realistic translation. Foremost is the challenge of data quality and representativeness: training datasets are typically small, unbalanced, and recorded with controlled laboratory conditions, while field samples illustrate considerable variability in terms of food matrix composition, temperature, humidity, background microflora, sensor-to-sensor variability, etc., which all poses the risk of inducing biases for AI-based models to generalise poorly. Second, model drift can occur with time due to sensor aging, biofouling, reagent lot changes, seasonal changes in contamination patterns, or changes in product uses or production practices that can lead to degraded predictive performance after deployment. Third, limited explainability and opaque decision making (black-box models) can limit user trust and also lead to difficulties when users attempt root-cause analysis when a system flags contamination. Fourth, field performance is typically less than bench performance as for example matrix interference, noise, and user handling variability can cause distributions to shift. Finally, the regulatory and validation barriers: AI/ML outputs need to be traceable, reproducible, and verifiable per specific protocols in order to be accepted for complying with testing requirements [182–185].

To address these issues, we have practical solutions such as: strong data governance (standardized metadata, controlled labelling, multiple site/multiple season datasets), domain adaptation and augmentation to improve the robustness of the matrix(es), continuous monitoring with drift detection and periodic re-calibration, “hybrid modelling” that combines physics/chemistry informed features with ML, uncertainty quantification with “reject options” for ambiguous samples, explainable AI (feature attribution for model building, model cards) for improved transparency, and strong field validation using pre-registered protocols as well as performance metrics that map onto regulatory expectations (sensitivity/specificity under real matrices, repeatability, inter-lab reproducibility, etc.) [182–185].

### Stability and Reproducibility of Biological Elements

A major limitation in the potential employment of biosensors engineered in the lab, for real-world usage is the limited stability and reproducibility of biological recognition elements (such as antibodies, enzymes, aptamers, and bacteriophages) [186]. Biological molecules can be inherently sensitive to environmental conditions such as, temperature, pH, ionic strength of solutions, storage conditions, and mechanical agitation. For example, antibodies used in immunosensors have the possibility of denaturing or losing some affinity due to conformational changes, while the enzymes used in an enzymatic biosensor may also denature over time by losing catalytic activity, resulting in the biosensor providing an inconsistent signal output [187, 188]. Such variability directly affects signal output, LOD, and shelf life, ultimately undermining standardization, scalability, and regulatory acceptance of biosensing platforms in food safety applications. Furthermore, batch-to-batch variability in biologically sourced materials, especially from polyclonal antibody production, adds another layer of unpredictability, impeding reproducibility across sensor lots and production runs [189, 190].

To address these issues, researchers have often utilized engineered or synthetic biorecognition approaches because they are more robust and consistent in performance. Recombinant monoclonal antibodies expressed in E. coli or CHO cells provide well-defined and reproducible binding kinetics, epitope specificity, scale-up production, and little inter-batch variability. Likewise, aptamers selected via SELEX with iterative thermal and chemical challenge rounds are more likely to yield thermodynamically stable and pH-tolerant sequences [191]. To further improve shelf life, immobilization techniques such as covalent grafting onto self-assembled monolayers, hydrogel entrapment, or cross-linking via glutaraldehyde are employed to prevent leaching and conformational drift of recognition elements. For bacteriophage-based biosensors, capsid stabilization using polyethylene glycol or lyophilization with sugar matrices has shown promise in preserving infectivity and targeting ability under ambient conditions [192].

From a material science perspective, the use of hybrid biorecognition elements, such as nanozyme-aptamer conjugates or peptide-functionalized metal-organic frameworks, is a promising frontier. These constructs mimic biological specificity while retaining synthetic durability. Moreover, integrating real-time self-calibration algorithms and internal standards in signal processing pipelines can mitigate the impact of minor variability in recognition performance. Lastly, AI-assisted design of aptamers and peptides, using generative models or structure-based docking, holds potential to not only optimize stability in silico but also predict degradation-prone motifs, guiding rational engineering of robust bioreceptors suitable for mass-scale deployment in food biosensing [193].

### Scalability, Cost, and User-friendliness

Also, despite great strides in regard to the sensitivity and specificity of biosensors, the issues related to the translation of laboratory prototypes into actual industry workhorse types remain a significant barrier. Generally, biosensors must deal with issues related to manufacturing scalability, cost of fabrication, and ease of use when put into the hands of the non-expert. Most high-performance biosensors contain materials such as gold nanoparticles, quantum dots, graphene, and intricate surface chemistry, as well as biological aspects that are simply not amenable to large scale, robust and reproducible fabrication. Furthermore, fabrication techniques such as photolithography, electron beam deposition, or precise microfluidic integration often require cleanroom infrastructure, skilled personnel, and extended turnaround times, factors that inflate production costs and hinder rapid commercialization. The unit economics of many biosensors thus remain unfavorable for widespread application in routine food testing, especially in low-resource or decentralized environments such as farms, slaughterhouses, or local food processing units [194].

Efforts to overcomes scalability and cost hurdles through the exploration of roll-to-roll printing, screen printing and 3D printing have been gaining interest as a key area for research focus. Mass-manufacturing biosensing components on paper or PET and PDMS, or other flexible substrates via low-cost additive manufacturing print methods reduce material waste and are compatible for printing electrode arrays, microchannels and detection zones at high-throughput low cost. Reducing biological reagent occupancy via droplet microfluidics of ultra-low volumes, or drying agents into spots via inkjet deposition enhance economic viability. Combined with biological reagent minimisation strategies (such as ultra-low volumes using droplet microfluidics or embedding dry-stable reagents via inkjet deposition), these strategies greatly improve the economic feasibility of biosensing systems. From a packaging viewpoint, lab-on-chip cartridges and point-of-care devices are now becoming available in plug-and-play architectures, loaded with pre-loaded reagents, and single-step sample loading mechanisms for less technical users to perform tests on the field with minimal risk of procedural errors [195].

The digital domain of smartphone-integrated biosensing platforms, with camera-based detection, cloud-enabled analytics, and user-guided mobile applications, is revolutionizing user accessibility by dispensing the need for cumbersome instrumentation. Novel paradigms for portable diagnostics are enabled by Bluetooth-enabled electrochemical readers, NFC-powered immunosensors, and fluorescence readouts using the smartphone camera with AI-assisted signal interpretation. Modular sensor platforms are being developed for further scale-up, allowing for the insertion of different sensor heads targeting different pathogens into a common readout system to distribute infrastructure cost over multiple assays. However, for true large-scale adoption, it is critical to ensure that these systems are compatible with regulatory frameworks and undergo rigorous validation under standardized food safety protocols (e.g., ISO 16140, AOAC). Incorporating design for manufacturability (DFM) early in the development phase, along with end-user co-creation, will be essential to balance performance, affordability, and usability in next-generation food biosensing technologies [196, 197].

### Toxicity, Stability, and Regulatory Barriers of Advanced Material

Despite their enormous performance advantages, the same emerging materials such as but not limited to nanomaterials, nanozymes, quantum dots, MXenes and advanced polymers all have a number of drawbacks that impede their direct use in food safety applications. The foremost limiting factor to the use of those emerging nanomaterials for any applications, especially food safety, is their toxicity. Concern of quantum dots based on heavy metals in particular manifest as contamination by leaching, bioaccumulation, and persistence when the particles are in contact with foods. Many nanomaterials also have long-term stability challenges such as aggregation, surface oxidation, loss of catalytic activity, and degradation of the functional coating under food-like fluctuating pH, temperate, and ionic conditions, leading to a drift in signal and loss of shelf life, and poor reproducibility - and aren’t suitable for routine or decentralized testing [198–200].

From a regulatory standpoint, the minimal toxicological data, absent standard protocols for material characterization, and ambiguous approval pathways further challenge the implementation of novel materials in biosensors. Typically, regulators want strong indication of safety, batch-to-batch reproducibility, and stability of sensor performance under real-world use conditions - parameters not fully satisfied by current lab demonstration levels. Information obtained from AI-assisted signal processing-based biosensors and novel nanocomposites face questions of traceability, interpretability, and validation against conventional reference methods. These challenges can be overcome through the design of inherently safer materials, effective encapsulation or immobilization strategies, long-term ageing (stability) tests and early-stage alignment with the requirements set by regulatory bodies (FDA, EFSA, FSSAI etc.) [201, 202].

### Environmental Effects on Biosensor Performance: Temperature and Humidity

Real environmental conditions, especially temperature and humidity, critically affect the biosensing performance of sensors in biosensing platforms due to their direct effect on biorecognition efficiency, signal transduction, and assay reproducibility. These variations can alter the binding kinetics between antibodies and antigens or between aptamers and targets, accelerate enzymes or nanozyme reactions, and cause partial denaturation in biological recognition elements, which contributes to signal drift or reduces sensitivity. Temperature in electrochemical biosensors affects solution conductivity, diffusion coefficients, and the processes of interfacial charge transfer, while in optical and colorimetric assays, it may alter the aggregation behavior of nanoparticles, fluorescence intensity, and background noise. These effects are normally negligible under controlled laboratory conditions but become more pronounced in real field environments, thus contributing to variability and reduced reliability when sensors are deployed across different stages of the food supply chain [203–205].

Humidity affects the performance of paper-based, polymeric, and portable biosensing formats mostly by influencing reagent hydration, capillary flow, and material swelling. These changes can result in poor development of signals coupled with false-negative or false-positive signal response. The biggest effect of high humidity is premature activation of reagents, while low humidity significantly slows down reaction kinetics or incomplete formation of the signal. Environmental moisture and temperature fluctuations have further effects on image acquisition, electronic stability, and long-term performance of smartphone and IoT integrated devices. Recent studies have emphasized protective encapsulation, moisture-barrier packaging, internal referencing or ratiometric sensing, and algorithm-based signal compensation to mitigate these effects. Environmental robustness should thus be incorporated into the design and validation of biosensors to ensure reliable performance under realistic food-testing conditions [87, 206].

### Comparative Biosensing Challenges Across Major Foodborne Pathogens

Although these three biosensing challenges - low pathogen concentrations, complex food matrices, and high specificity - are common for E. coli, Salmonella spp., and Listeria, the basic causes and practical consequences are very different for the pathogens. Very low infectious doses, particularly for the Shiga toxin-producing variants, along with frequent associations with fresh produce and water, put extremely high demands on the limits of ultra-low detection and matrix tolerance of sensing [207–209]. A large number of serovars in the case of Salmonella, belonging to almost all food categories, enhance problems such as cross-reactivity, multiplex discrimination, and enrichment bias. In contrast, Listeria monocytogenes has high environmental persistence, biofilm formation, and exceptional proliferation capabilities at refrigeration temperatures, thus shifting biosensing challenges toward surface monitoring, cold-chain surveillance, and viability-aware detection. Fully appreciating both shared and pathogen-specific constraints underpins the design of biosensors that will be analytically sensitive and context-appropriate for real-world food safety monitoring [210]. A comparative overview of pathogen-specific biosensing challenges, highlighting shared and distinct constraints across *E. coli*, *Salmonella*, and *Listeria*, is summarized in Table 5.

**Table 5:** Comparative biosensing challenges for major foodborne pathogens

Challenge Dimension	E. coli (esp. STEC O157:H7)	Salmonella spp.	Listeria monocytogenes	Implications for Biosensor Design
Typical infectious dose	Very low (10–100 CFU)	Moderate	Low–moderate (high-risk groups)	Requires ultra-low LOD
Common food matrices	Produce, water, dairy, meat	Poultry, eggs, nuts, processed foods	RTE foods, dairy, deli meats	Matrix-specific pretreatment
Environmental persistence	Moderate	Moderate–high	Very high (biofilms)	Surface & biofilm sensors needed
Growth at refrigeration	No	Limited	Yes (psychrotrophic)	Cold-chain monitoring
Strain diversity	Moderate	High (many serovars)	Lower (species-focused)	Multiplex & selectivity control
Viability relevance	High	High	Critical	RNA/phage/metabolic sensing
Enrichment dependency	Often required	Frequently required	Often required	On-chip enrichment
Regulatory risk	Outbreak-driven	High incidence	High mortality	Pathogen-specific validation

### Regulatory Hurdles and Standardization Issues

The regulatory and standards space for biosensors in food safety is immature and disjointed, which impedes success in real-world applications. Biosensors have demonstrated excellent analytical capabilities in the laboratory; however, there are serious complications to transitioning biosensors to legally acceptable diagnostic and detection methods for foodborne pathogens due to the fragmented and obscure approval paths. Directionally, one of the biggest obstacles continues to be the absence of universally accepted methods for validating biosensor performance for a diverse range of pathogens, food matrices, and environmental characteristics. Regulatory agencies like the U.S. FDA, EFSA, and Codex Alimentarius often demand rigorous multi-lab validations, ISO-compliant reproducibility studies (e.g., ISO 16140), and comparisons against gold-standard culture-based methods, all of which are time-consuming, costly, and technically demanding for academic labs or small-scale developers [211].

Another important aspect lies in the potential that the biosensor outputs may no longer be consistent with regulatory thresholds and threshold-based decision making. While traditional analysis results in CFUs or PCR cycle threshold values, biosensor outputs are typically in relative units (current, fluorescence intensity, absorbance ratios, etc.). These do not line up very easily with one another, making the regulatory benchmarking challenging. For biosensors in conjunction with AI/ML algorithms, extra scrutiny over model interpretability, training dataset diversity, software validation are all needed - and currently, there’s no consensus guidance out there from food safety regulators either. The dynamic and adaptive nature of AI platforms makes certification tricky as traditional validation relies on the timeless and static nature of the products being examined leaving little room for surprises [189, 212].

To overcome these regulatory constraints and bottlenecks, a number of targeted approaches are beginning to emerge. These centre on establishing harmonised validation frameworks to evaluate biosensors (AOAC Performance Tested Methods and ISO 23418 for rapid molecular methods that we have discussed before); the clear aim is to ease the path to acceptance and standardisation of evaluating biosensors against “classic” methods. An internal calibration standard, and combination modalities of biosensor with output traceable to CFU with confidence, will close the gap on new signal and existing measures. On the AI side, regulatory consistency of ML models can be supported through the use of XAI and locked model architectures with version control, ensuring reproducibility and facilitating regulatory review. We can support regulatory review with libraries of reference materials and libraries of biosensor challenge panels containing real food samples spiked with pathogen loads. Pre-regulatory validation under realistic conditions will assist in the validation process. Even early and sustained engagement with regulators during sensor design as opposed to after development are increasingly recognized as valuable to expedite approval and ensure that biosensors meet analytical and regulatory needs [213–215].

## Conclusion, Future Prospects, and Research Directions

The persistent threat of Escherichia coli, Salmonella spp., and Listeria monocytogenes to food safety globally underscores the need for fast, reliable and field-applicable biosensing approaches. In the last decade there had been remarkable developments and optimizations of biosensors based on electrochemical, optical and microfluidic principles also with the introduction of novel materials including nanostructures, aptamers, nanozyme, and 2D platforms. The contribution of AI and ML to multiplicative benefits for schematics requiring pattern recognition, real time snapshots of incoming data, and decision making in biosensors have allowed for greater gains to be made in terms of biosensor performance and capability. Examples of this across various matrices of food products has proven the usefulness of AI-assisting detection systems in terms of sensitivity, time savings, and complex feature learning. Making progress towards deploying some form of biosensing in the real world for on-site pathogen surveillance and forewarning of contamination events.

Looking forward, biosensing platforms should evolve from isolated innovations toward integrated, interoperable and scalable systems. New platforms must move toward self-powered biosensors leveraging triboelectric or biofuel cell mechanisms for battery-free operation as well as modular multi-analyte sensors capable of concurrently detecting multiple pathogens and toxins in a single run. Also, transfer learning, federated learning and explainable AI can tackle data scarcity and improve model transparency - two elements vital for regulatory approval and trust (and wider adoption) of AI-based food diagnostics. IoT-enabled biosensors connected to blockchain platforms may also lead to tamper-proof traceability and real-time data sharing across food supply chains, creating a network of connected safety technologies rather than static testing tools. Another emerging area is the integration of wearable or embedded sensors in food packaging and processing lines to enable continuous “farm-to-fork” reliability and monitoring.

However, for this vision to be achieved, future work will have to resolve the remaining bottlenecks through multi-disciplinary convergence, such as: (i) robust biological recognition elements that have longer shelf-life and are tolerant to matrix variability, (ii) standardised validation protocols, regulatory alignment frameworks, but yet are cost-effective and large-scale manufacturable; and (iv) greater focus on user-centred design to maximise non-expert operability. Ultimately, the goal is to establish next-generation biosensors that are intelligent, scalable, affordable, and regulation-ready, thereby empowering a safer, smarter, and more resilient global food system.

## Data Availability

Data will be made available based on a reasonable request.
